# Isolation of Cancer Stem Like Cells from Human Adenosquamous Carcinoma of the Lung Supports a Monoclonal Origin from a Multipotential Tissue Stem Cell

**DOI:** 10.1371/journal.pone.0079456

**Published:** 2013-12-04

**Authors:** Jennie P. Mather, Penelope E. Roberts, Zhuangyu Pan, Francine Chen, Jeffrey Hooley, Peter Young, Xiaolin Xu, Douglas H. Smith, Ann Easton, Panjing Li, Ezio Bonvini, Scott Koenig, Paul A. Moore

**Affiliations:** 1 MacroGenics, Inc., South San Francisco, California, United States of America; 2 MacroGenics, Inc., Rockville, Maryland, United States of America; Mayo Clinic College of Medicine, United States of America

## Abstract

There is increasing evidence that many solid tumors are hierarchically organized with the bulk tumor cells having limited replication potential, but are sustained by a stem-like cell that perpetuates the tumor. These cancer stem cells have been hypothesized to originate from transformation of adult tissue stem cells, or through re-acquisition of stem-like properties by progenitor cells. Adenosquamous carcinoma (ASC) is an aggressive type of lung cancer that contains a mixture of cells with squamous (cytokeratin 5+) and adenocarcinoma (cytokeratin 7+) phenotypes. The origin of these mixtures is unclear as squamous carcinomas are thought to arise from basal cells in the upper respiratory tract while adenocarcinomas are believed to form from stem cells in the bronchial alveolar junction. We have isolated and characterized cancer stem-like populations from ASC through application of selective defined culture medium initially used to grow human lung stem cells. Homogeneous cells selected from ASC tumor specimens were stably expanded *in vitro*. Primary xenografts and metastatic lesions derived from these cells in NSG mice fully recapitulate both the adenocarcinoma and squamous features of the patient tumor. Interestingly, while the CSLC all co-expressed cytokeratins 5 and 7, most xenograft cells expressed either one, or neither, with <10% remaining double positive. We also demonstrated the potential of the CSLC to differentiate to multi-lineage structures with branching lung morphology expressing bronchial, alveolar and neuroendocrine markers in vitro. Taken together the properties of these ASC-derived CSLC suggests that ASC may arise from a primitive lung stem cell distinct from the bronchial-alveolar or basal stem cells.

## Introduction

The cancer stem cell hypothesis states that cancers arise from mutational or epigenetic changes in tissue stem (or progenitor) cells, which allow these cells to escape intrinsic and extrinsic growth controls and become invasive [1]. In addition to the strong evidence supporting this hypothesis in hematopoetic cancers, there is a growing body of evidence that a number of solid tumors, including brain, colon, breast and lung [2] are hierarchically organized with a subset of self-renewing stem-like cells. Furthermore, there is recent direct evidence from animal models that colon, brain, and skin cancers can arise from tissue stem-like cells present in the adult tissues [3-5]. The stem-like characteristics of self-renewal, tumorigenicity, drug resistance, and the ability to recapitulate all of the cell types of the tumor, have allowed investigators to address important questions concerning the biology of this cell population, which bear directly on patient treatment [3,6].

There is evidence from both animal models and human disease that lung cancers are among those that arise from stem-like cells [7-10]. However, the source of stem cells (SC) involved in normal lung development, maintenance, and repair following injury is somewhat more complicated than tissues such as the skin or colon (for reviews see: [11,12]), where multiple “conditional” stem cells have been thought to be involved in the repair of different portions of the lung after injury [13]. These may, or may not, be the same stem cells responsible for tissue maintenance [14]. It has been suggested that within lung cancers, NSCLC (non small cell lung cancer) adenocarcinomas arise from SC at the bronchial alveolar junction (BASC), squamous carcinomas arise from basal SC of the bronchi and trachea, and small cell carcinomas arise from pulmonary neuroendocrine cells [11,12]. 

Adenosquamous carcinoma (ASC) of the lung is an infrequent subtype of NSLC cancer diagnosed by tumors that contain both cells with squamous and adenocarcinoma (AC) histological phenotypes (defined as >10% of each). ASC comprises 4-8% of NSCLC, but is very aggressive so that patients with ASC have a poorer prognosis that those with either squamous cell or adeno-carcinomas [15]. It has been hypothesized that these tumors arise either from mixtures of cells derived from the two tumors of different types, the further mutation of one type giving rise to the other, or a monoclonal origin where both derive from a common, as yet unidentified, precursor [16-19]. Studies on ASC tumors from patients have shown that cells from the adeno and squamous portions of a tumor have similar, but not necessarily identical, chromosomal abnormalities [16], or mutations [17] suggesting a monoclonal origin for this type of tumor. However, this does not constitute proof of the ability of these two phenotypes to arise from a single cell, and the cell from which ASC might originate remains unidentified.

In this report, we describe cancer stem like cells (CSLC) isolated from patients with ASC using defined serum-free culture conditions. Furthermore, we demonstrate that these cells have the properties of self-renewal, tumorigenicity, and metastasis. Tumors derived from these cells, designated LUCA22 and LUCA35, including those derived from cloned single cells and from metastases contained both components with glandular (adeno) differentiation and areas of squamous differentiation, supporting a monoclonal origin for this tumor. The CSLC growth properties, gene expression and the ability to form branching structures in 3D co-culture with lung stroma further support the hypothesis that these CSLC have stem cell like properties. Additionally, the xenografts derived from these CSLC contain cells positive for chromagranin A, Mucin 5A, vimentin, surfactant protein D, aquaporin 5 and cytokeratins 5, 7, 14 and 20, demonstrating the ability of these cells to undergo multi-lineage differentiation. These data suggest that the ASC are monoclonal in origin and possibly arise from a primitive lung stem cell distinct from the bronchiolar alveolar stem cell (BASC) or the basal stem cell of the upper airways.

## Materials and Methods

### Ethics statement

Lung cancer tissues were obtained through the National Disease Research Interchange, 1880 John F. Kennedy Boulevard, 6th Floor, Philadelphia, PA 19103 (url: http://ndriresource.org/). Institutional IRBs approved the NDRI protocols for tissue acquisition and use, and appropriate informed consent was obtained from patients by NDRI. No information was available that would in any way compromise the anonymity of the patients. This study was carried out in strict accordance with the recommendations in the Guide for the Care and Use of Laboratory Animals of the National Institutes of Health. The MacroGenics West Institutional Animal Care and Use Committee approved all protocols using mice. All surgery was performed under anesthesia, and all efforts were made to minimize suffering. Animals were checked daily and sick animals removed and euthanized. All animals were euthanized at the end of the experiment before tissue collection. Euthanasia was by CO2 asphyxiation delivered in a compressed gas cylinder. 

### CSLC selection, expansion, and characterization

Lung cancer tissues were obtained through the National Disease Research Interchange, 1880 John F. Kennedy Boulevard, 6th Floor, Philadelphia, PA 19103. Institutional IRBs approved the protocols for tissue acquisition and use, and appropriate informed consent was obtained from patients by NDRI. The lung cancer derived cell lines were expanded from ASC and AC tumors (or tumor stroma) and master and working cell banks prepared. Short tandem repeat (STR) analysis was used for identification. 

The defined conditions appropriate for normal human fetal lung tissue epithelial stem/progenitor cells [20] were used initially and then optimized empirically for isolation and growth of cancer tissue as previously described [21]. Serum free conditions were derived that enrich for, and allow expansion of, a small population of cells from ASC and AC (but not squamous cell carcinoma (SCC)) of the lung. Addition of 1-2% serum to the hormone supplements or supplementation with high 10% (v/v) serum resulted in a non-tumorigenic population of cells with limited growth potential *in vitro* and the properties of stromal cells. See Methods S1 and Table S1 for detailed methods for isolation of cells from tumors; and defined media and supplement concentrations for selection, expansion, cloning and differentiation of the ASC-CSLC; and expansion of stromal cells. The conditions for the differentiation of lung stem cells in 3 dimensional Matrigel^TM^ cultures were modified from Delgado, et al as described in the Methods S1. The tumor samples and isolated CSLC lines were commercially characterized by their unique Short Tandem Repeat patterns using 16 STR regions. This analysis is described in Methods S1 and summarized in Table S2. 

 The tumor type and stage (from the pathology reports) of the 4 tumor CSLC and 3 stromal CSLC cultures are summarized in Table S3. Five ATCC cell lines, derived from AC, SC and ASC using serum-containing media, were used as controls in a number of experiments. The characteristics of these lines are summarized in Table S4

### Genetic analyses

#### Reverse transcriptase (RT-PCR) analysis

DNA was isolated from animal samples using the Wizard SV Genomic DNA Purification kit following the manufacturer’s protocol (Promega). Human DNA was quantified by PCR using human-specific RPL19 gene primers and probes [22]. The primers were purchased from SA Biosciences as “RT^2^ qPCR Primer Assays”. The list of primers and catalog numbers is given in Table S5. To evaluate expression of specific genes, RT-PCR reactions were performed on cDNA generated from total RNA isolated from the individual CSLC cultures using RT² qPCR Primer Assays and *GAPDH* (glyceraldehyde 3-phosphate dehydrogenase) as a constitutively active gene control, and RT² SYBR® Green/ROX qPCR Mastermix (Qiagen). Expression of selected genes was also determined in normal human lung and isolated normal human bronchial epithelial cells. Threshold Cycle (Ct) for each primer set was determined by running RT-PCR reactions in the ABI PRISM® 7000 Sequence Detection System (Life Technologies Corporation). DCt for each ISC gene was determined as (Ct[gene]- Ct[GAPDH]), then Relative Expression to GAPDH determined as 2^-DCt^.

#### Short tandem repeat (STR) analysis

Sixteen-locus short tandem-repeat (STR) analysis was performed on the original tumor specimens, if sufficient material was available (7/9 cases), on the master and working cell banks, and longitudinally at several intervals on each line, and on tissues excised from mouse tumor xenografts to evaluate identity. STR Analysis was performed with the AmpFℓSTR® Identifiler® PCR Amplification Kit (Applied Biosystems, Foster City CA). Amplified loci were loaded into Applied Biosystem’s 3730xl Automated Sequencer and analyzed using Applied Biosystem’s GeneMapper software version 4. MSI-H was diagnosed by the presence of allelic instability at more than 5 of 15 autosomal STR loci [23].

#### Mutation analysis

Mutation analysis on the cell lines was carried out by two complementary methods.  Analysis of *KRAS* exon 2, *BRAF* exon 15, *PIK3CA* exons 9 and 23, and the Mutation Cluster Region (MCR) of *APC* exon 15 was performed by sequencing amplified genomic DNA as previously described [24,25]. The MALDI-TOF mass spectrometry platform and OncoCarta panel were used to detect mutations at 238 sites in 19 gene loci, including those above, as previously described [26]. 

### Flow cytometry analysis

Cultured cells were removed with collagenase/dispase (Roche Applied Science) or trypsin/EDTA (Invitrogen), washed, and re-suspended in F12/DMEM medium (Gibco/Invitrogen) + 1.0% BSA (Rockland Immunochemicals). Cell counts were obtained utilizing Guava ViaCount reagents (Guava Technologies). Fifty thousand viable cells were aliquoted into round-bottom, low binding HTS 96-well plates (Beckton Dickinson), incubated with antibody at 4°C for 20 min., washed in analysis buffer, and counter-stained, when necessary, with 2 µg/mL GaM-IgG (H+L) conjugated with Alexafluor532 or PE (Invitrogen). Cells were washed and either fixed in analysis buffer + 0.1% formaldehyde (Polysciences), or re-suspended in analysis buffer, then analyzed on a Guava-PCA96 or a FACScan (Becton Dickinson). Data was analyzed using FlowJo software (TreeStar Inc.). Results were calculated as binding intensity (log_10_ [stained] – log_10_ [isotype control]). See Methods S1 for additional details. ALDH was assessed using the flow based ALDEFLUOR^®^ assay (Stem Cell Technologies) [27] with the substrate titrated from 1:10 to 1:100 dilution.

For double-immunostaining analysis of CK5/7 binding, confluent plates of cells were dissociated with 0.05%Trypsin/EDTA, neutralized with soybean trypsin inhibitor, and washed by centrifugation. The resulting pellet was fixed in 4% paraformaldehyde (PFA) followed by permeablization in 0.1% Triton-X 100. Primary antiserum was added at a 1:50 dilution of mouse anti-human cytokeratin 7 (EPR1619Y) (Abcam, Ad68459), added at the same time as 0.5µg/ml of mouse anti-human cytokeratin 5 (BioCare Medical, # PM234AA). Secondary antiserum was 1µg/ml goat anti-rabbit Alex Fluor 488 (Life Technologies™) added concurrently with 1µg/ml goat anti-mouse R-Phycoerytherin (Life Technologies™). Unstained samples, secondary-only samples, and single primary sample controls were used in the analysis. 

### In vivo tumorigenicity

CSLC lines were implanted under the renal capsule (SRC) of immune deficient NOD SCID common γ chain receptor knockout mice (NSG) mice as collagen-embedded cells as previously described [22,28] and allowed to grow for up to 32 weeks. Animals were examined at 2-8 months for tumors and metastases. Tumors were removed and embedded for IHC, or separated to single cells and used for PCR or marker analysis as indicated. 

### Immunohistochemistry

#### Fixed tissues

Excised tumor xenograft tissue was fixed in 10% neutral formalin (Sigma), embedded into a paraffin block and 5μm sections cut. Slides were de-paraffinized and rehydrated, pretreated with antigen retrieval solution (citrate buffer pH 6) in a decloaking chamber (Biocare Medical), and stained for 1 hour with indicated anti-human antibodies (e.g. CD34, CD44) using the supplier’s protocol (Biocare Medical). Slides were rinsed with PBS and incubated for 30 minutes with goat anti-mouse HRP (MACH2, Biocare Medical) and diaminobenzidine chromogen substrate, then lightly counterstained with hematoxylin and eosin. 

#### Frozen tissues

For frozen sections human normal lung and lung cancer, LUCA-derived xenografts, cell pellets from 2D cultures, or organoids from 3D culture were embedded in O.C.T, then cryostat sectioned at 7 µm. Serial sections were fixed in 4°C acetone for 10 minutes, and air-dried for 30 minutes. Slides were incubated in 3% H_2_O_2_ for 10 minutes followed by two rinses in buffer. 5% normal goat serum was applied to the slides for 10 minutes then blown off. Slides were incubated with the test antibodies (concentrations and incubation times determined by previous optimized protocols) and washed twice with buffer. Controls were incubated with an isotype control antibody corresponding to each experimental antibody tested. Following primary antibody incubation, the slides were incubated with Dako Envision HRP anti mouse or rabbit polymer for 30 minutes followed by two rinses in buffer. Diaminobenzidene tetrahydrochloride (DAB) was used as a chromogen to visualize the staining.

#### Immunofluorescence on monolayer cultures

Cells were grown on either glass coverslips or glass chamber slides (Lab-Tek), then fixed with 4% PFA and permeabilized with 0.1% TritonX-100. Primary antiserum was 1µg/ml of mouse anti-human cytokeratin 5 (BioCare Medical, # PM234AA), added at the same time as a 1:100 dilution of rabbit anti-human cytokeratin 7 (EPR1619Y) (Abcam, Ad68459). Secondary antiserum was 2µg/ml goat anti-mouse Alex Fluor 488 (Life Technologies™) added concurrently with 2µg/ml goat anti-rabbit Rhodamine Red™-X (Life Technologies™). Mouse IgG1 was used as an isotype control, and secondary-only conditions were used for non-specific staining controls. Immunostaining was visualized on a Nikon TE300 microscope with an ET Sedat Quad Filter Set and a Retiga EXi CCD camera. iVision software was used to capture images.

## Results

### LUCA CSLC characterization

Tumor tissue, shipped on ice, was received from a wedge resection of primary lung tumors. Tissue was dispersed and cells plated in serum-free conditions (or media containing serum for the expansion of stromal cells). This defined medium selected and expanded a small proportion of epithelial cells from 3 of 4 lung adenocarcinomas (AC) (LUCA 32, LUCA33) and 2 of 2 adenosquamous carcinomas (ASC) (LUCA22, LUCA35) tissues. In contrast, no successful cultures were obtained from squamous carcinoma tissue samples (4 separate attempts) using this same medium. The addition of serum at the initiation of the primary cultures from the tumor results in the early expansion of stromal cells in 3 of 3 instances (e.g. LUCA11 (1% serum + hormone additives), LUCA36 (10% serum)), which can be expanded for limited passage and banked. These stromal cells have a fibroblastic appearance in 10% FBS and are not tumorigenic when implanted at 5x10^5^ under the sub-renal capsule of an immunodeficient mouse. 

### STR Characterization and mutational analysis of tumor-derived cell cultures

Short tandem repeat (STR) analysis at 16 sites gave a unique pattern for each of the 6 lines, which were distinct from each other and from any ATCC lines (see Table S2). Three of 4 lines showed some loss of heterozygosity when comparing the tumor sample (which would include non-tumor cells) and the CSLC. Some gain of heterozygosity was seen with extended passage (p47= >150 population doublings).

Mutational analyses revealed that 100% of the ASC (LUCA22) CSLC exhibits a single G12V point mutation within the coding regions of *KRAS*, with no mutations found in *APC* or *PIK3CA*. The AC lines (LUCA32 and LUCA33) also had a single point mutation in KRAS only (G12V and G12C respectively) (Table S3). 

### Tumorigenicity and metastases

**Figure 1:**
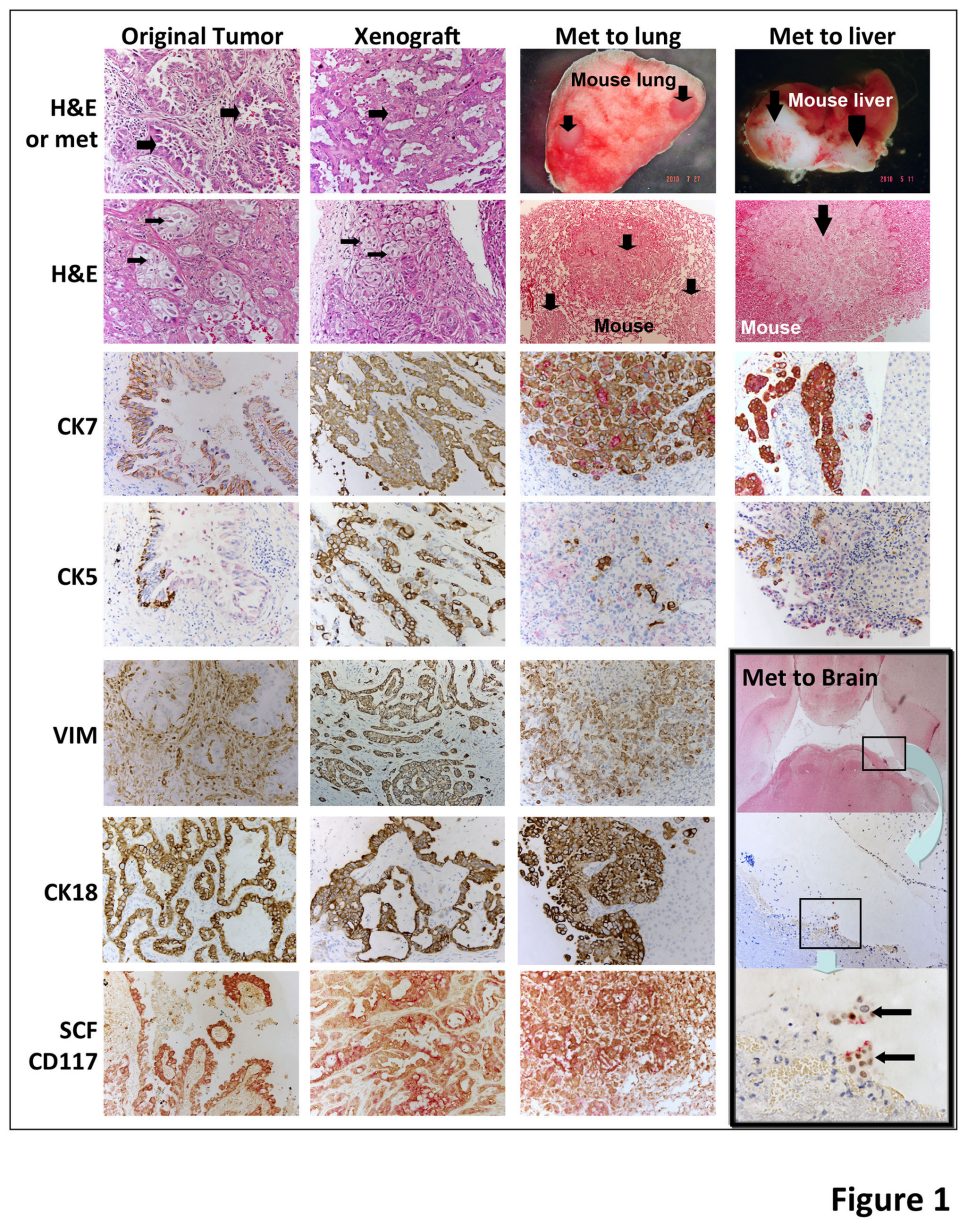
Histopathology of patient tumor and xenografts derived from CSLC. Comparison of morphology and histopathology of the original patient tumor from which LUCA22 was derived and xenografts and metastases derived from the LUCA22 CSLC. Sections of the tumors are stained with H&E to show adenocarcinoma (thick arrows) and squamous (thin arrows) morphologies (top 2 rows, left). Top right 2 rows shows metastases to the lung and liver (arrows) and H&E sections of these organs showing metastatic nodules (arrows). Additional sections are stained for cytokeratins 5 (CK5), 7 (CK7) and 18 (CK18), or vimentin (VIM). The bottom row is double stained for stem cell factor (SCF-brown) and c-kit (CD117 (red). The inset box on the lower right shows increasing magnifications of human LUCA22 cells (box, arrows) that have metastasized from a sub renal tumor to the brain, stained for the lung tumor specific Napsin (red)/TTF1 (brown) double stain.

To test for tumorigenic potential, LUCA22 cells at various inocula (5x10e4, 5x10e3, or 5x10e2) were embedded in collagen buttons and implanted under the sub-renal capsule (SRC) of severely immune deficient NSG mice. Tumors were observed at all cell inocula by 31 weeks (Figure 1, Table S6). At higher cell inocula metastases were observed in multiple organs as early as 10 weeks after SRC implantation. The metastases increased in distribution and size with time with all animals inoculated with the highest cell number showing metastases at 16 weeks or longer. Metastases were seen in the liver, spleen, pancreas, mesentery, diaphragm and, occasionally, at distant sites such as the lung and brain (Figure 1). Cells inoculated subcutaneously grew into tumors but did not metastasize (5x10e5, up to 24 weeks).

**Figure 2:**
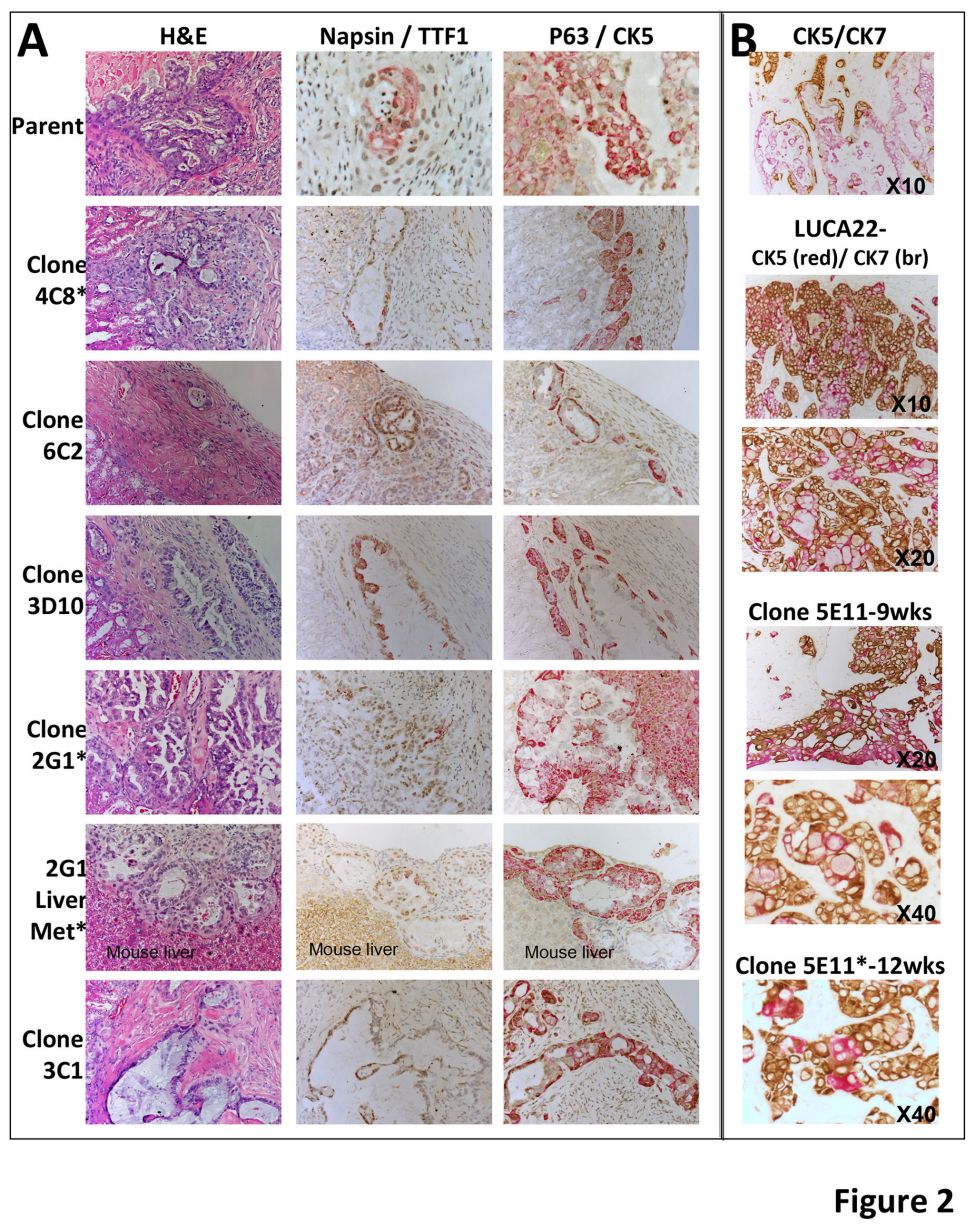
Histopathology of clonal CSLC-derived xenografts. The cloned LUCA22 cells give rise to xenografts staining as adeno- and squamous carcinoma. Xenografts arising from the LUCA22 CSLC, 5 LUCA22 clones, and a metastasis from clone 2G1 are shown stained with H&E, Napsin/TTF1, or p63/CK5 after 8 weeks in the animal (A). Clones that metastasized are indicated by *. B. Xenografts derived from the patient tumor (top) of the LUCA 22 and xenografts derived from LUCA22 clone 5E11 at the times and magnifications indicated. These sections are double stained for CK5 (red) and CK7 (brown).

One element defining CSCs is that they can form a tumor from a single cell and recapitulate the morphology of the original patient tumor. To test whether a single cell could give rise to differentiated tumors, eight single cell clones derived from LUCA22 were chosen for expansion and implantation in the SRC. Tumors grew from all 8 clones selected for testing (Figure 2, Table S6). Two of the clones showed metastases by 8 weeks *in vivo*. Thus, the LUCA22 line is capable of giving rise to a metastatic tumor from a single cell. We next compared the xenografts derived from LUCA22 ASC to the original patient tumor using IHC to look at markers characteristic of ASC.

### Comparative analysis of patient and xenograft tumors by IHC

Since CK5 and CK7 expression in different portions of the same tumor is pathognomonic for ASC, we compared the distribution of CK5 and CK7 staining within the original patient tumor, xenografts derived from LUCA22 cells, LUCA22 clones, and metastases from these xenografts. LUCA 22 derived xenografts appeared as a poorly differentiated ASC that closely resembled the histology of the patient’s original tumor (Figure 1). Some areas of the tumor had the appearance of an adenocarcinoma and were positive for CK7 staining, while other areas of the tumor had features of a squamous carcinoma and stained positively for CK5, thus reproducing the ASC phenotype seen in the original patient tumor. Even tumors derived from 500 cells exhibited the ASC pattern of both CK5 and CK7 staining (data not shown). Metastases to the lung and liver also contained cells that were both positive for CK5 and CK7 staining (Figure 1).

To further characterize these tumors, we stained the tissues with two antibody combinations used by pathologists to differentiate lung adenocarcinomas (Napsin A + TTF1) [29] and squamous cell carcinomas (p63+CK5) [30]. These two sets of antibodies were observed to stain different areas of the parental tumor and all 8 clonally derived xenografts and metastases (Figure 2 A, Figure S1). Clonally derived xenografts, double stained for CK5 and CK7, also were expressed in distinct, but adjacent regions of the tumor (Figure 2 B). Interestingly, the patient tumor, as well as xenografts, expressed CK18, an epithelial marker, and vimentin, a mesenchymal marker (Figure 1), with the stromal portion of the patient tumor, but not the xenograft, also staining positively with the anti-human vimentin antibody. 

Additionally, we examined the tumors for stem cell factor (SCF) and it’s receptor CD117 (c-KIT) and ALDH1A1 [9], proteins reported to play a role in lung tumorigenesis. Both the patient’s tumor and the xenograft tumors were positive for both SCF and CD117, found in adjacent areas of the tumor (Figure 1) although SCF staining was more extensive than CD117 staining (Figure S2 A). ALDH1A1 also was found in both the patient tumor and xenografts derived from LUCA22 as well as metastases to the lung (Figure S2 A,B). The LUCA22 CSLC were positive for ALDH activity, a portion of which could be neutralized by the ALDH1 specific inhibitor DEAB (Figure S2 C)

### Cytokeration expression in CSLC

**Figure 3:**
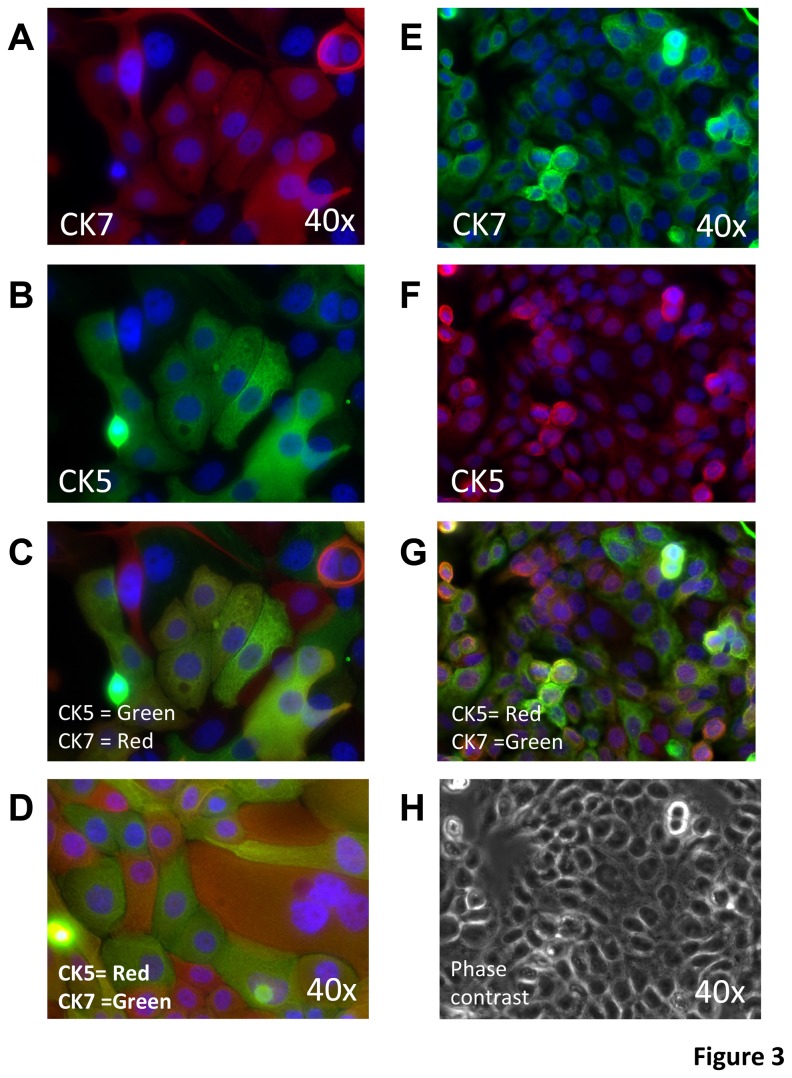
Staining of ASC CSLC monolayers for CK5 and CK7. Immunofluorescence (panels A-G) and phase contrast (H) of permeabilized monolayer cultures stained for CK5 (red), CK7 (green), or both. LUCA22 (A-C) the LUCA22 clone 5E11 (D) and LUCA35 (E-G) are shown. Panels A-C and E-G show the same field individually stained and the overlay of both. Panel H shows the same field as (E-G) as a phase contrast image. All images are 40X magnification.

**Figure 4:**
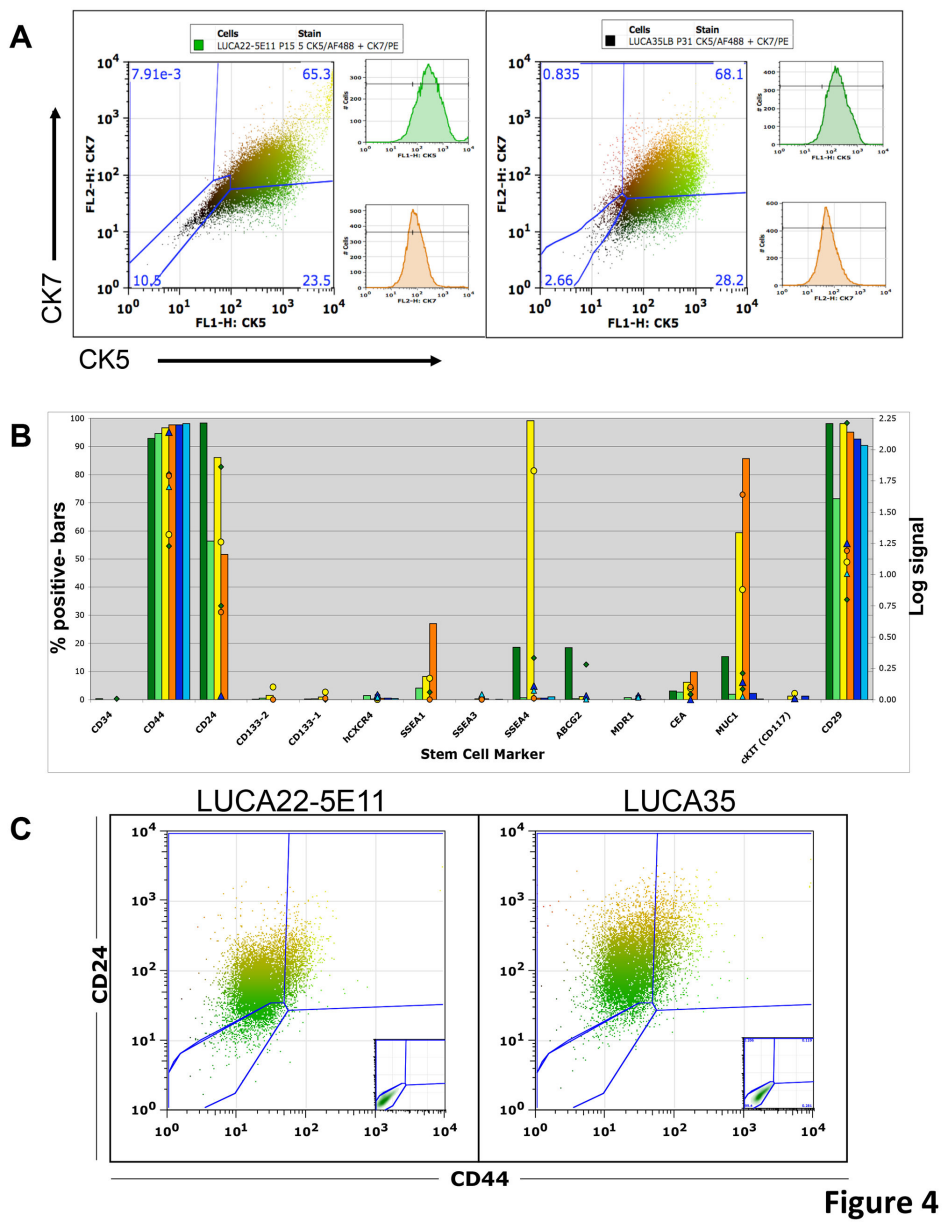
Analysis of protein expression on lung cancer derived cells. Panel A is data from flow analysis of permeabilized LUCA22 clone 5E11 and LUCA 35 ASC cells double stained for CK5 and CK7. The % positive cells and log signal from flow analysis of cell surface proteins (B) is shown for the 2 ASC-derived CSLC (LUCA22 (dark green bars), LUCA35 (light green)), the 2 AC-derived CSLC (LUCA32 (yellow), LUCA33 (orange)) and lung cancer stromal cells (LUCA11 (dark blue), LUCA36 (light blue)) in panel B. The log binding (symbols) and percent binding over isotype control (bars) is shown for each. Panel C shows a single population that dual labels with CD44 and CD24 antibodies for the LUCA22 and LUCA35 cells.

Since the expression of both CK5 and CK7, within different areas of the tumor, is characteristic of ASC we double stained LUCA22 and LUCA35 CSLC monolayers for CK5 and CK7 using rhodamine or FITC labeled antibodies. Surprisingly, most CSLC cells were double positive CK5+CK7+. The doubly stained cells seemed to have variable levels of CK5 and CK7 in the cells (Figure 3). This was also true of the clonally derived line 5E11 (Figure 3 D). Quantitation of CK5 and CK7 double stained cells was performed using flow cytometry of permeabilized, fixed and stained cells (Figure 4 A). Again, the LUCA 22, cloned LUCA22 -5E11 and LUCA35 cells all were predominantly double positive for CK5 and CK7. As controls, four ATCC serum-derived cells lines were double stained for IHC and flow analysis (See Results S1). None showed this double staining pattern of the CSLC. Interestingly, when the LUCA22 CSLC (including single cell clones) are allowed to form tumors the CK5 and CK7 expression sorts into different cell populations (Figure 2 B). Thus there are CK5+/CK7-, CK5-/CK7+, and a small population of CK5+/CK7+ cells in the tumors (Figure S4). The large CK5-/CK7- population seen in the dispersed SQ and SRC tumors may be contaminating murine stromal cells, since we did not actively select against these cells for this analysis.

### Cell surface marker analysis

To further characterize the populations selectively derived from lung cell tumors, cells from the 4 CSLC lines (ASC: LUCA22, LUCA35; AC: LUCA32, LUCA33) and 2 stromal lines (LUCA11, LUCA36) were analyzed by flow cytometry for the binding of a panel of antibodies to human markers often used to select for, or identify, CSLC from solid tumors (Figure 4 B). CD44 and CD29 were present on all lines, including the stromal lines. CD24 was present on all 4 of the CSLC lines, but not the stromal cells. None of the lines bound either of the CD133 monoclonal antibodies that have been reported to select for CSC in some tumors, although the universality of CD133 as a stem cell marker in lung cancers has been challenged [12]. Mucin 1 (MUC1) and stage specific embryonic antigen 1 (SSEA1) were expressed at a higher level on the AC lines than the ASC lines while ABCG2 is found on LUCA22, but not the other lines. To look for homogeneity of the populations, the LUCA22-5E11 clone and the LUCA35 cell line were double labeled for CD24/CD44 (Figure 4 C). The double labeling showed that one population bound both antibodies. Four clones of LUCA22 were selected and analyzed in parallel with the LUCA22 parental line. Binding was very similar for the clones and parental line for most markers with unimodal distributions, although some variation in peak binding was observed. (Figure S5 A-C). Since the stem cell marker CD117 was found on only a small portion of the CSLC, we attempted to enrich for this population using FACS. After 4 successive sorts, we were unable to increase the percentage of cells positive for CD117, suggesting that these cells do not represent a separable subpopulation of cells in the CSLC (Figure S5 D).

### Gene expression in CSLC lines

**Figure 5:**
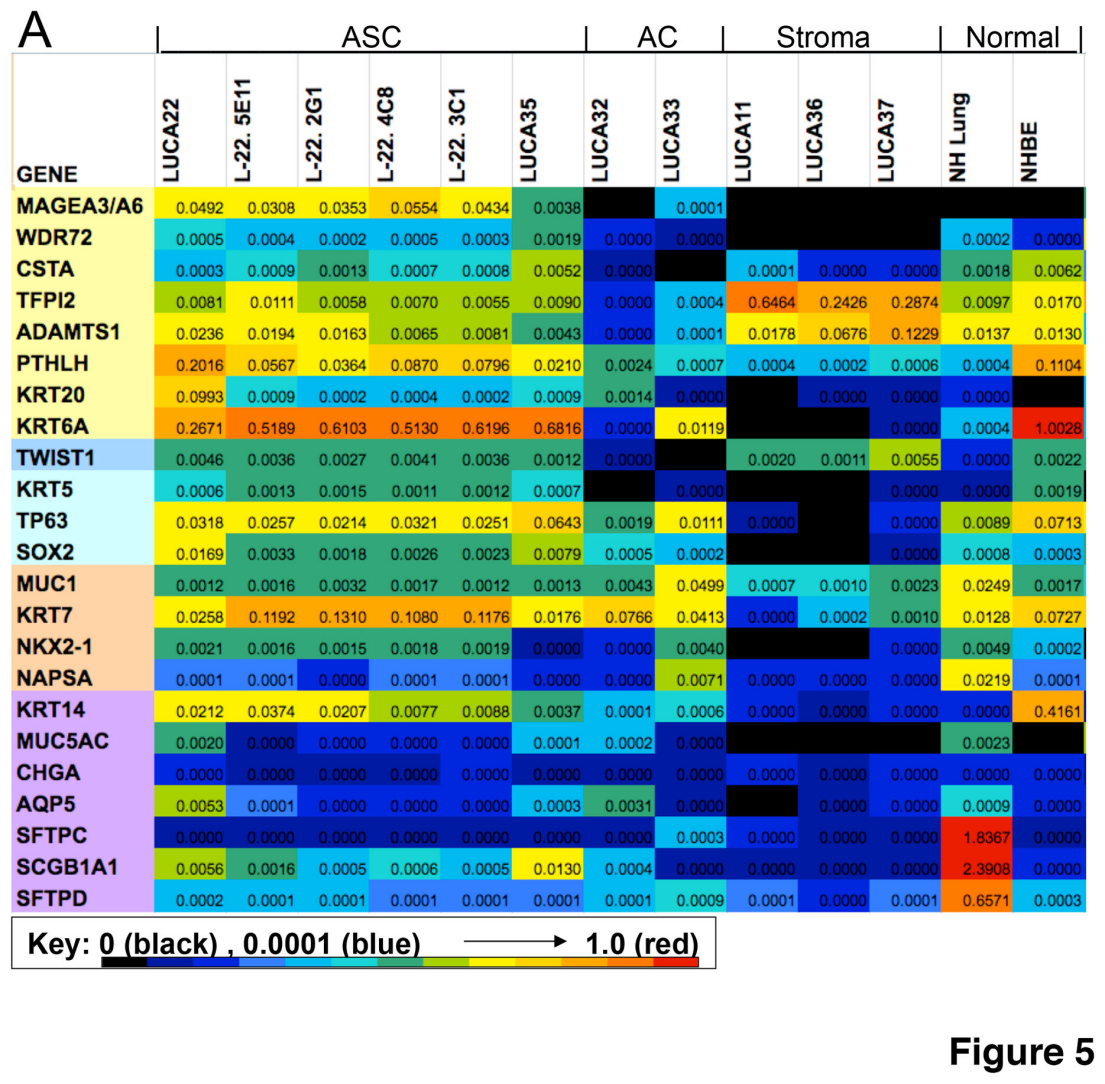
Gene expression in ASC. Panel A compares RTPCR gene expression in ASC and AC CSLC compared to normal lung (NHL), normal human bronchial epithelial cells (HBE) and ATCC cell lines (A) (see Table S3). Panel B compares the expression of the same gene set in CSLC grown as usual (2D) or in Matrigel and differentiation media alone (3D) or co-cultured with stromal cells (3DC). For comparison tumor cells isolated from xenografts (XD) are shown. The data was generated using QTPCR with probes specific for the mRNAs shown (see Results S1). In A, the probe sets highlighted in yellow are genes over expressed in ASC vs. AC CSLC, blue shows marker gene for SC, peach for AC and those sequences highlighted in purple are genes expressed in differentiated lung cells. Expression levels are color coded from negative (black), low in blues, to highest in red.

To more extensively characterize gene expression in these CSLC, we looked at a set of 23 genes selected from those reportedly expressed in AC and SCC lung cancers and differentiated normal lung cell types by RTPCR. The expression pattern of the ASC lines was unique in that they co-expressed (including clones) multiple genes thought to be characteristic of AC and SCC, as well as a number of normal lung lineage markers. The expression of a number of genes was strongly up-regulated in the ASC lines compared to the AC lines (Figure 5) including: *MAGEA3, MAGEA6, CSTA, TFPI2*, *KRT5, TWIST1* and *PTHLH*. Of these, only the MAGEA3/A6 expression was entirely restricted to lung cancer cells (>30X higher in ASC than AC) and not seen at all in the tumor stroma or normal lung samples. Markers for other differentiated cell types were expressed at low levels in the CSLC (*SCGB1A1*, Clara cells; *MUC5AC*, goblet cells; *AQP5*, type I pneumocytes; and *SFTPC*, BASC) or at very low levels (*SFTPD*, type II pneumocytes; *CHGA*, neuroendocrine cells). As expected, all of these genes that are markers of differentiation were expressed in the control RNA from either normal human lung (NH Lung) or human bronchial epithelial cells (HBE). 

We next looked at the mRNA levels for these and other genes that could be used to differentiate between AC, SC, and ASC. As shown in Figure 5, cytokeratin 5 mRNA (*KRT5*) was highly expressed in the ASC-CSLC (LUCA22, LUCA35), but not in the AC-CSLC or stromal cells (LUCA32, LUCA33). Cytokeratin 6A (*KRT6A*, [31]) was also strongly expressed in the ASC compared to the AC and absent in the stroma. Genes associated with squamous cell cancer, *KRT5*, cystatin A (*CSTA*) [32] tumor protein p63 (*TP63*) [30] and *SOX2* [33], were expressed at higher levels in ASC, but also expressed to some extent in the AC. In contrast, *KRT7* was expressed in all CSLC, but not in the tumor stroma. Cytokeratin 14 (*KRT14*), a basal cell marker, was seen at high levels in HBE, in the ASC CSLC, and at much lower levels in the AC CSLC and tumor stroma. There was p63 (*TP63*) message expression in all lines, except the stroma, but it was most strongly expressed in the ASC-CSLC. Napsin (*NAPSA*) was expressed in only one of the 2 AC, while TTF1 (*NKX2-1*) was strongly expressed in one AC, and expressed at lower levels in one ASC line, neither of these tumor markers was present in the stroma. These data support the observed IHC staining of TTF1 and p63 in the LUCA 22 pellet (Figure S1), and are consistent with the co-localization of CK5 and CK7 in the LUCA22 and LUCA35 CSLC. 

Three of the genes that were strongly up-regulated in ASC-CSLC compared to AC-CSLC (*CSTA, TFPI2*, and *ADAMTS1*) were also expressed in the stromal cultures and in the normal bronchial epithelium. Other genes expressed predominantly in the ASC-CSLC lines include, PTH-like hormone (*PTHLH*), *TWIST1*, and WD repeat domain (*WDR72*) (Figure 5). The expression profiles of the 4 clonally derived LUCA22-CSLC tested, and the LUCA22 parental line were very similar (Figure 5). Expression of the gene panel was variable in the 5 ATCC control cell lines derived from lung tumors in serum containing medium, as shown in Figure S3.

### Differentiation in vitro

**Figure 6:**
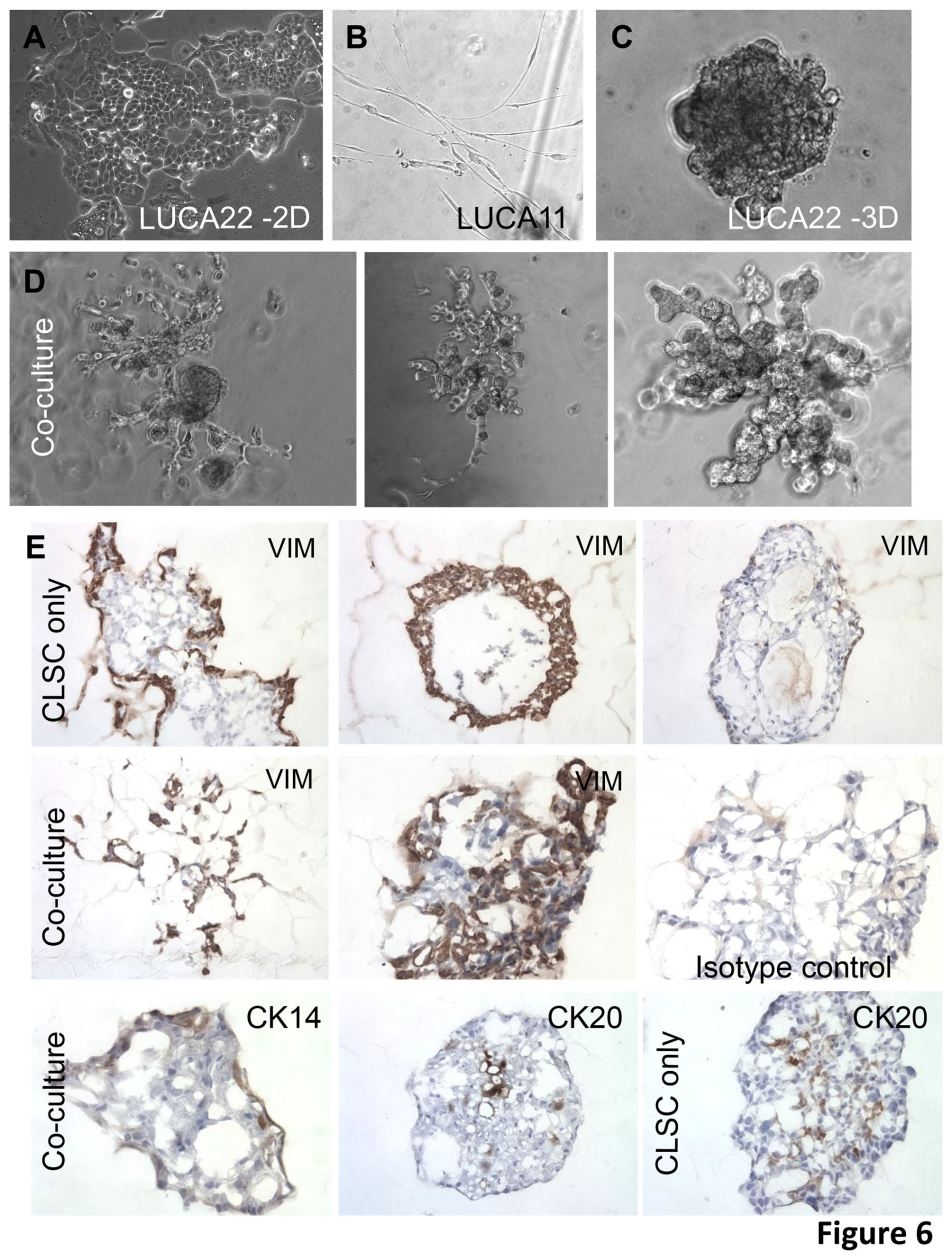
Differentiation of LUCA22 CSLC to organoids in three dimensional Matrigel cultures. LUCA22 cells were cultured as described in a 3D Matrigel + hormones (“3Diff” condition) alone or mixed with LUCA11 stromal cells before plating. Phase contrast photos show the LUCA22 monolayer cultured on fibronectin (A); the LUCA11 cells plated alone in 3Ddiff (B); or LUCA22 CSLC alone in 3Ddiff (C). The mixture of LUCA11 and LUCA22 cells formed branching three-dimensional structures as seen in the 3 panels in D at 14 days of culture. At 14 days the cultures were embedded and frozen for sections. Antibody isotype controls were used on every other section although only a few are shown for comparison. Vimentin, CK14 and CK20 staining is shown in the indicated panels in E for the CSLC alone, or in co-culture with stromal cells.

In order to better understand the differentiation potential of LUCA22, and perhaps, to shed light on the cell type in the lung giving rise to ASC, we explored the ability of these cells to differentiate *in vitro*, in a 3 dimensional (3D) culture system modified from Delgado, et al. [34] (Figure 6). The CSLC cells in Matrigel 3D culture with additional “differentiation” hormones (see Table S1) made round cellular aggregates similar to those previously described by Delgado et al. with their transformed human bronchial epithelial-derived (HBE) stem cells (Figure 6 C). When the LUCA22 cells were suspended in Matrigel in the LUCA growth medium, however, they rapidly died. If stromal/ fibroblastic cells derived from lung tumor tissue were placed in the SF growth medium in Matrigel they tended to attach to the plate beneath the Matrigel and spread, or remained as suspended single cells, but did not grow (Figure 6 B). However, if the two cell types were combined, and then suspended in Matrigel in the differentiation medium, extensive 3-dimensional branching structures were formed. These structures were embedded in OCT and sectioned for further analysis. Xenografts were frozen, sectioned and stained as differentiated tumor controls. Antibody binding specificity was validated using normal human tissues, which served as positive controls for each marker (images not shown) (Figure 6 D). 

**Figure 7:**
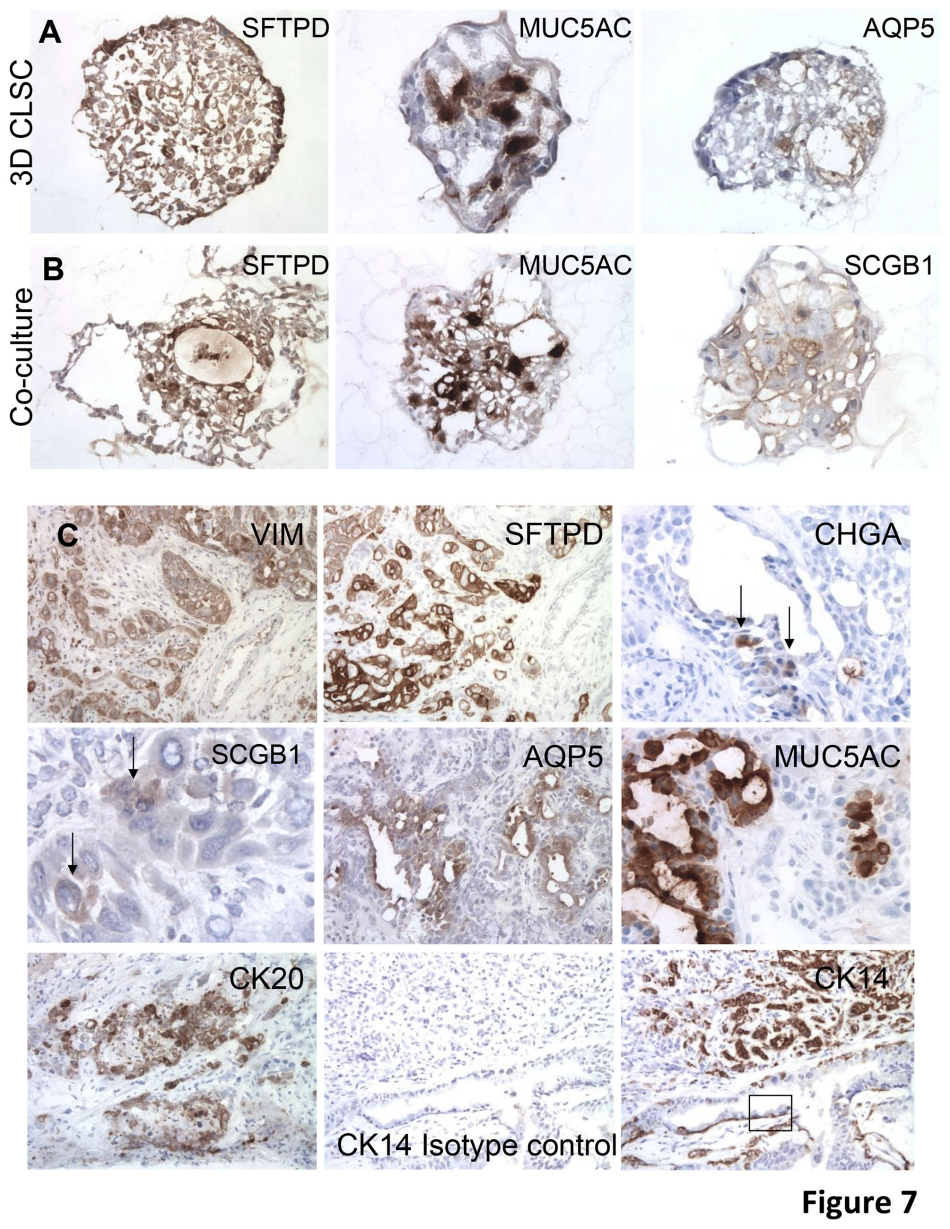
Comparison of expression of lung lineage markers in 3D culture and xenografts derived from LUCA22. LUCA 22 CSLC were grown in 3D cultures (A), co-plated with stromal cells in 3D cultures (B), or differentiated in vivo in xenografts (C) and stained for lung lineage markers. Staining for the lineage markers SFTPD, CHGA, MUC5A, AQP5, and SCBG1 are shown for the 3D cultures in panels A & B, as indicated. The 9 panels in show the LUCA 22 xenografts stained for the same markers and additionally stained for vimentin and cytokeratins 20 and 14 (shown for 3D cultures in Figure 6). The box in the CK14 stained panel indicates a portion of the xenograft where the staining is limited to basal cells.

We examined sectioned CSLC-derived differentiated organoids for the expression of genes normally expressed by differentiated sub-types of adult lung cells. The human tumor-derived stromal cells and the xenograft stroma (host derived) were both vimentin positive. Interestingly, both single LUCA22 CSLC and co-cultured CSLC stained for vimentin although the pattern of staining was different, with some organoids in the 3D single culture staining strongly for vimentin and some negative. The co-cultured 3D cells tended to be more uniformly vimentin positive, especially the outlaying structures (Figure 6 E). The CSLC cell pellet stained uniformly for CK14 (not shown) but the staining was reduced in the 3D cultures while in some areas of the xenografts only the basal cells in bronchial-like structures were CK14 positive (Figure 6 E, Figure 7). CK20 stain was sporadic in the 3D cultures. Both 3D cultures were uniformly positive for surfactant protein D (SFTPD) while both the cultures and xenografts were negative for SFTPA (Table 1), although control sections of normal lung pneumocytes stained positively with the antibody used. Both CSLC and 3D cultures contained cell staining for MUC5A. With LUCA22, the co-cultures, but not the CSLC 3D cultures, were weakly positive for Clara cell secreted protein (SCGB1); while the opposite was true for aquaporin 5 (AQP5) (Figure 7). Xenografts were positive for SFPTD, AQP5, VIM, and MUC5a as well as scattered cells positive for the neuroendocrine marker chromagranin A (CHGA) (Figure 7, Table 1). The LUCA35 CSLC cultured in 3D and xenografts gave similar results as those for LUCA22 (Table 1, Figure S6). A human specific smooth muscle actin antibody stained only the human stromal cells (Table 1).

**Table 1:** Immunohistochemistry of marker protein expression.

**Marker gene**	***KRT14***	***SCGB1A1***	***AQP5***	***SFTPA***	***SFTPD***	***MUC5AC***	***CHGA***	***ACTA2***	***VIM***	***KRT5***	***KRT7***	***KRT20***
**Lit-Cell Type**	BBC	Clara	PN-I	PN II	PN II	BE	PNE	Stroma	CSLC cells	SC	AC	mucBACs
**HPA cell type**	BBC+PN	BE	N/A	BE+ PN	PN+M	BE	No stain	No stain	PN	BE	BE+PN	NO STAIN
**HPA- lung CA**	SC + AC	No stain	N/A	AC	AC	SC 1/23	No stain	AC + SC	AC	SC	AC	SC 1/45
**LUCA11/36 – 2D** (**stromal**)	**-**	**-**	**-**	**-**	**-**	**-**	**-**	**+**	**+**	**-**	**-**/+F	**-**
**LUCA22 2D** (**ASC**)	**+**	**-**	**-**	**-**	**+**	**-**	**-**	**-**	**+**	**+**	**+**	**+** **F**
**LUCA22 3D**	**+**	**-**	**-**	**-**	**+**	**+**	**-**	**-**	**+**	**+**	**+**#2	**+**
**L22+L113D**	**+**	**-**	**-**	**-**	**+**	**+**	**-**	**-**	**+**	**+**	**+**#1	**-**
**LUCA35 2D** (**ASC**)	**+**	**+**	**+**	**-**	**+**	**+**	**-**	**-**	**-**	**+**	**+**	**+** **F**
**LUCA35 3D**	**+**	**+F**	**+**	**-**	**+**	**+**	**-**	**-**	**-**	**+**	**+**#2	**-**
**L35+L113D**	**+**	**+F**	**+**	**-**	**+**	**+**	**-**	**-**	**+** **P**	**+**	**+**#2	**-**
**LUCA22** Xenograft	**+**	**±**	**+**	**-**	**+**	**+**	**+**	**+**	**+**	**+**	**+**	**+**
**LUCA35** Xenograft	**+**	**+**	**+**	**-**	**+**	**+**	**+**	**SM**	**+** **1-2%**	**+**	**+**	**+**

Notes: The top 3 rows summarize the data from the literature (Lit) and the human protein atlas (HPE) on the presence of the marker in specific cell or tumor types: BE= bronchial epithelium; BBC= bronchial basal cells; PN= pneumocyte; PNE=neuroendocrine; AC adenocarcinoma; SC squamous cell carcinoma. Staining is seen in: F= few cells (<1%); P= peripheral cells; SM= murine smooth muscle in tumor; #1= some cells double stained CK5+/CK7+; #2= most cells double stained CK5+/CK7+.

## Discussion and Conclusions

Lung cancer remains one of the most frequent and lethal cancers. Lung neoplasms are histologically diverse, making tumor classification difficult [35]. Normal lung tissue also is comprised of a mixture of multiple specialized epithelial and stromal cell types, providing a number of potential stem or progenitor cells that could possibly be transformed leading to cancer. This may, in part, explain the various distinct types of lung cancer that arise. Indeed, mouse models suggest that AC arise from bronchial alveolar stem cells [BASC] in the lung [10], while SC are thought to arise from basal stem cells in the tracheal and upper bronchial epithelium [11]. Stem-like cells have been isolated from normal lung tissue [34] and human lung tumors using markers to enrich for subsets of cells [7,11]. It has been suggested that, given the presence of multiple stem / progenitor cells in the lung, the method of isolation, as well as the starting tumor type, might well influence which type of CSLC is isolated and the resultant properties exhibited [36,37]. 

A peculiar subtype of lung cancer is the adenosquamous carcinoma (ASC), which comprises a minority of lung tumors (4-8%) and is associated with poor prognosis [15-17]. ASC includes areas of squamous histology, expressing the squamous cell markers CK5 and p63, and areas of adenocarcinoma (AC) morphology expressing markers such as CK7, Napsin, and TTF1. Since squamous carcinoma (SCC) is thought to arise in the upper respiratory tract and AC to arise in the bronchial alveolar region, the origin of the ASC tumors is unclear. Various hypotheses for the derivation of ASC have been suggested: ASC may arise as an admixture of the 2 tumors (collision tumor); as AC with squamous metaplasia; as primarily SCC evolving to ASC [19], or from a bipotential undifferentiated cell (monoclonal origin) [16]. Two studies have used micro-dissection of the adeno- and squamous carcinoma portions from individual ASC tumors and compared DNA specific patterns between the two. In one instance loss of heterozygosity (LOH) was observed at a highly polymorphic trinucleotide CAG repeat in the X-linked human androgen receptor region (HUMARA) [16]. In this study, the AC and SCC components showed a monoclonal pattern of LOH in all 4 specimens examined whereas the adjacent normal tissue did not show LOH, supporting a common clonal origin of both AC and SCC components. Other studies [17,38,39] tested for mutations of the EGF-R and KRAS genes in micro dissected AC and SCC portions of patients with ASC and demonstrated identical mutations in both components, again suggesting monoclonality. Finally, Kanazawa et al [19] reported the presence of the same p53 mutation in both components of all 12 tumors with p53 over expression. In 3 of 4 tumors with chromosomal abnormalities, the abnormality was shared by both the AC and SCC components. These results, together with additional IHC for specific protein expression, led them to conclude that ASC arises during monoclonal transition from SCC to AC. 

Our results demonstrate that the CSLCs isolated from ASC patients recreate the original ASC morphology when implanted in immune deficient (NSG) mice at low cell numbers, or as inocula grown from single cell clones. All eight single cell clones tested also made tumors containing both AC and SCC components. Interestingly the xenografts in the sub renal capsule metastasize and all metastases also show both the adeno- and squamous components. Additionally, while the majority of the CSLC double stain for CK5 and CK7, the majority of the cells in the differentiated xenograft (and the original tumor) are CK5+/CK7-, CK5-/CK7+, or CK5-/CK7- (stromal cells). These results strongly support a monoclonal origin for ASC, and suggest that this tumor originates from a single cell that expresses both squamous and adeno- carcinoma markers and has the capacity to differentiate to either AC-like or SC-like areas, rather than one component arising from the other.

The individual LUCA22 and LUCA35 cells stained positively, at variable levels, for both CK5 and CK7. Intriguingly, they also expressed mRNA for, and protein staining for, markers of multiple cell types in the adult lung including basal cells, Clara cells, bronchial epithelial cells, pneumocytes, and neuroendocrine cells. Cells expressing each of these markers were found in the xenografts derived from the ASC-CSLC. A co-expression of multiple markers (albeit a different set of markers) has also been reported with murine [10] and human [34] bronchial epithelial stem cells. The expression of multiple markers in the CSLC again supports the hypothesis that the AC and SCC portions in the tumor arise from a single precursor cell rather than one type evolving from the other. This interpretation is supported by the work of Bastide, et al. [40] who examined the gene expression patterns of ASC arising in a rat radiation-induced lung cancer model. They concluded that ASC lung tumors are more complex than simple mixes of AC and SCC components, with neuroendocrine differentiation and ERK pathways preferentially deregulated in the ASC compared to AC and SCC. Interestingly, we also observed the expression of chromagranin A (CHGA), a neuroendocrine marker, in LUC22 and LUCA35 derived xenografts. 

The LUCA22 and LUCA35 ASC stem-like cell populations, including those arising from a single cell clone, also express markers of both AC and SCC tumors, and additionally express several mRNAs associated with epithelial mesenchyme transition [41] including vimentin, TFPI2, ADAMS1, and TWIST1. Low-level expression of genes associated with normal basal cells (KRT14), goblet cells (MUC5A), Clara cells (SCGB1A1), and pneumocytes (SFTPD, AQP5) is seen in the CSLC monolayer cells, the 3D cultured cells and the xenografts. We additionally show a strong preferential expression of the MAGEA3/A6 gene in the ASC. This gene is expressed only early during human development in the embryoid bodies and the nervous system, and in the normal adult testis [42]. MAGEA expression has been reported in NSCLC [43], and esophageal cancers [44] and may play a role in drug resistance [45]. 

The properties of the ASC-derived CSLC fulfill the biological criteria for cancer stem cells in that they can self renew, form tumors from a cloned single cell and re-create the original tumor phenotype, including the appearance of both squamous and adenocarcinoma portions of the tumor. Our ability to isolate and perpetuate ASC-derived CSLC in culture allows us to expand and study this rare tumor CSLC, while being able to re-create xenografts mimicking the patient tumor at will. Furthermore, cells cultured alone or co-cultured with tumor stromal cells made organoids with elaborate three-dimensional branching structures. The ASC CSLC are distinct from transformed bronchial epithelial stem-like cells (BASC) of Delgado et al. [34] in that they expressed a different set of markers. Both CSLC express p63, K14, and surfactant protein D (SP-D), although the HBE-BASC of Delgado, et al. express more AQP5 and Clara cell markers than the CSLC, and uniquely express SP-A and SP-C. In contrast, the LUCA22/35 cells uniquely express MUC5A and neuroendocrine markers. The LUCA22/35 cells are null for CD133 and CD34, in contrast to the CD133+, CD34+ cells isolated by Eramo, et al. [7] from NSCLC lung tumors followed by growth as tumor spheres, again suggesting that the ASC CSLC described here have a unique phenotype. The difference in expression patterns of stem-like cells isolated by 3 different methods from lung tumors supports the contention of Kim et al. [36,37] that the mode of isolation used may bias to outcome towards different populations of CSC in the complex set of NCSCLC. 

Co-expression of multiple lineage markers, and the dual staining of CSLC for CK5 and CK7 and the expression of other genes characteristic of both SCC (SOX2, KRT6A, p63) and AC (MAGEA3, Muc1, TIFF, and NAPSA), suggest that these unique ASC CSLC derive from a bi- or multi-potential lung stem or progenitor cell, which may be distinct from the BASC cell or CK14+ basal SC, and that adenosquamous carcinomas have a monoclonal origin from these cells. 

## Supporting Information

Figure S1 **Staining of cells and xenografts using lung cancer pathology diagnostic stains.** The Napsin/ TTF1 stain (A) is diagnostic for lung adenocarcinoma while the CK5 / p63 is suggested for squamous cell carcinoma (B). Napsin and p63 are nuclear stains. A low power CK5 and CK7 stain of another xenograft is shown for comparison. Cells are removed from the plate with trypsin, pelleted, fixed, sectioned and stained.
(TIF)

Figure S2 **IHC staining and ALDH1A1 activity of xenografts and CSLC.** The LUCA22 xenograft stained for CD117 and SCF are shown in panel A. The LUCA22 xenograft and patient tumor double stain for ALDH1A1 (brown) and CK5 (red) (A). A metastasis to the lung is shown stained for ALDH1A1 (B). LUCA22 CSLC have a low level of non-inhibited ALDH1A1 activity by flow analysis (C, green line).
(TIF)

Figure S3 **Double staining of ATCC cell lines for cytokeratins 5 and 7.** Fixed permeablized monolayers of SKMES (a squamous cell carcinoma line) A549 (AC), and 2 ATCC ASC lines, H596 and H647 are shown. RT-PCR Gene expression analysis of the panel of genes analyzed for the CSLC in Figure 5 is shown for 5 ATCC cell lines derived from AC, ASC, and SCC tumors (see Table S4). The flow analysis of H596 and H647 double stained for CK5 and CK7 are shown in B.
(TIF)

Figure S4 **Analysis of CK5 and CK7 protein expression by flow cytometry.** CK5 and CK7 protein expression was further analyzed in lung cancer derived cells by flow analysis of permeabilized LUCA22 monolayer cells double stained for CK5 and CK7 (2D). For comparison, human tumor cells isolated from subcutaneous (SQ) or sub-renal (SRC) implanted LUCA22 cells after 20 weeks in vivo are shown. Xenografts were dispersed to single cells for analysis and contain both stromal (CK7-/CK5-) and tumor cells.
(TIF)

Figure S5 **Flow analysis of cell surface proteins in 5 clones.** Five randomly selected LUCA 22 clones were analyzed for expression of cell surface proteins using tagged antibodies and flow. These were compared to the parental LUCA22 line (A). The biggest variability was seen in SSEA4 expression shown as separate histograms in B. Double stain of 4 clones for CD24 and CD44 are shown in C. The diagrams in D show staining for CD117 (& side scatter) after 1 bulk sort and after the 4^th^ successive sort of the population. The CD117 remains a minority population.
(TIF)

Figure S6 **The LUCA35 cells and xenografts express multiple lung cell markers.** Cells were grown in the usual medium (LUCA35 2D, F-H top) in Matrigel with differentiation medium (LUCA35 3D, F-H bottom) or in vivo as xenografts (9 panels in I). Frozen sections were stained as indicated. All isotype control stains on adjacent sections were negative (see CK20 and isotype control section, bottom center for an example).
(TIF)

Methods S1 **CSLC selection and expansion from NSLC tumor tissue.** LUCA CSLC characterization.
Differentiation in vitro.
(DOC)

Results S1 Cytokeratin staining of ATCC control cell lines. Analysis of LUCA22 clones. Use of double stains for SC and AC.
(DOCX)

Table S1 **Media supplements for selective growth and differentiation of lung tumor derived cells.**
(DOCX)

Table S2 **STR analysis of lung cancer-derived cell lines.**
(DOCX)

Table S3 **Characteristics of Lung Tumor-derived cell lines.**
(DOCX)

Table S4 **Characteristics of ATCC lung cancer cell lines.**
(DOCX)

Table S5 **“RT^2^ qPCR Primer Assay” probe catalog numbers.**
(DOCX)

Table S6 **Tumorigenicity of LUCA22 and clones in SRC model.**
(DOCX)
