# Supplementary figures and images for: Isolation of Cancer Stem Like Cells from Human Adenosquamous Carcinoma of the Lung Supports a Monoclonal Origin from a Multipotential Tissue Stem Cell
Source: PLoS One. 2013 Dec 4;8(12):e79456. doi: 10.1371/journal.pone.0079456 (PMC3850920; doi:10.1371/journal.pone.0079456)

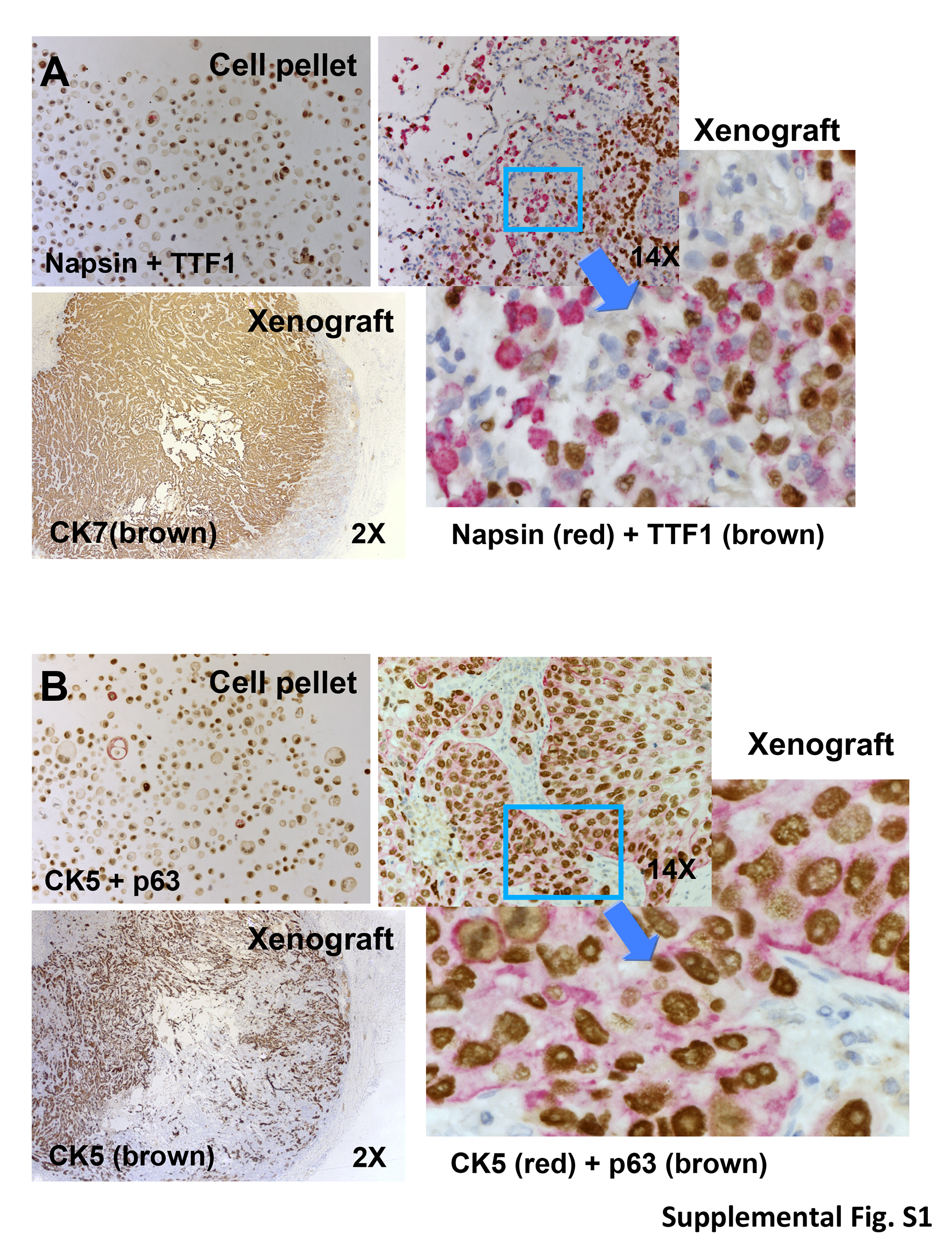

Supplement: Figure S1 — Staining of cells and xenografts using lung cancer pathology diagnostic stains. The Napsin/ TTF1 stain (A) is diagnostic for lung adenocarcinoma while the CK5 / p63 is suggested for squamous cell carcinoma (B). Napsin and p63 are nuclear stains. A low power CK5 and CK7 stain of another xenograft is shown for comparison. Cells are removed from the plate with trypsin, pelleted, fixed, sectioned and stained. (TIF) [file pone.0079456.s001.tif]

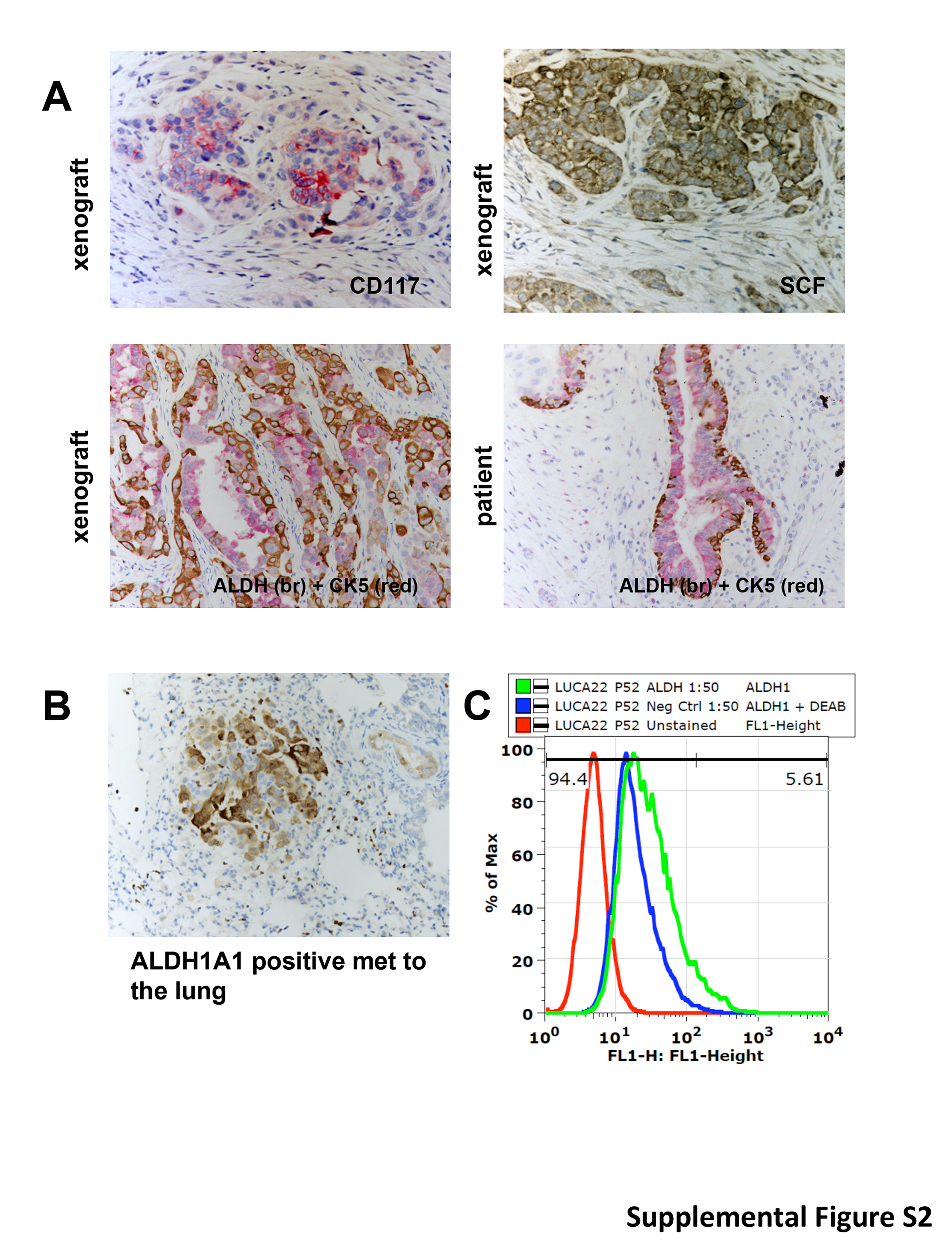

Supplement: Figure S2 — IHC staining and ALDH1A1 activity of xenografts and CSLC. The LUCA22 xenograft stained for CD117 and SCF are shown in panel A. The LUCA22 xenograft and patient tumor double stain for ALDH1A1 (brown) and CK5 (red) (A). A metastasis to the lung is shown stained for ALDH1A1 (B). LUCA22 CSLC have a low level of non-inhibited ALDH1A1 activity by flow analysis (C, green line). (TIF) [file pone.0079456.s002.tif]

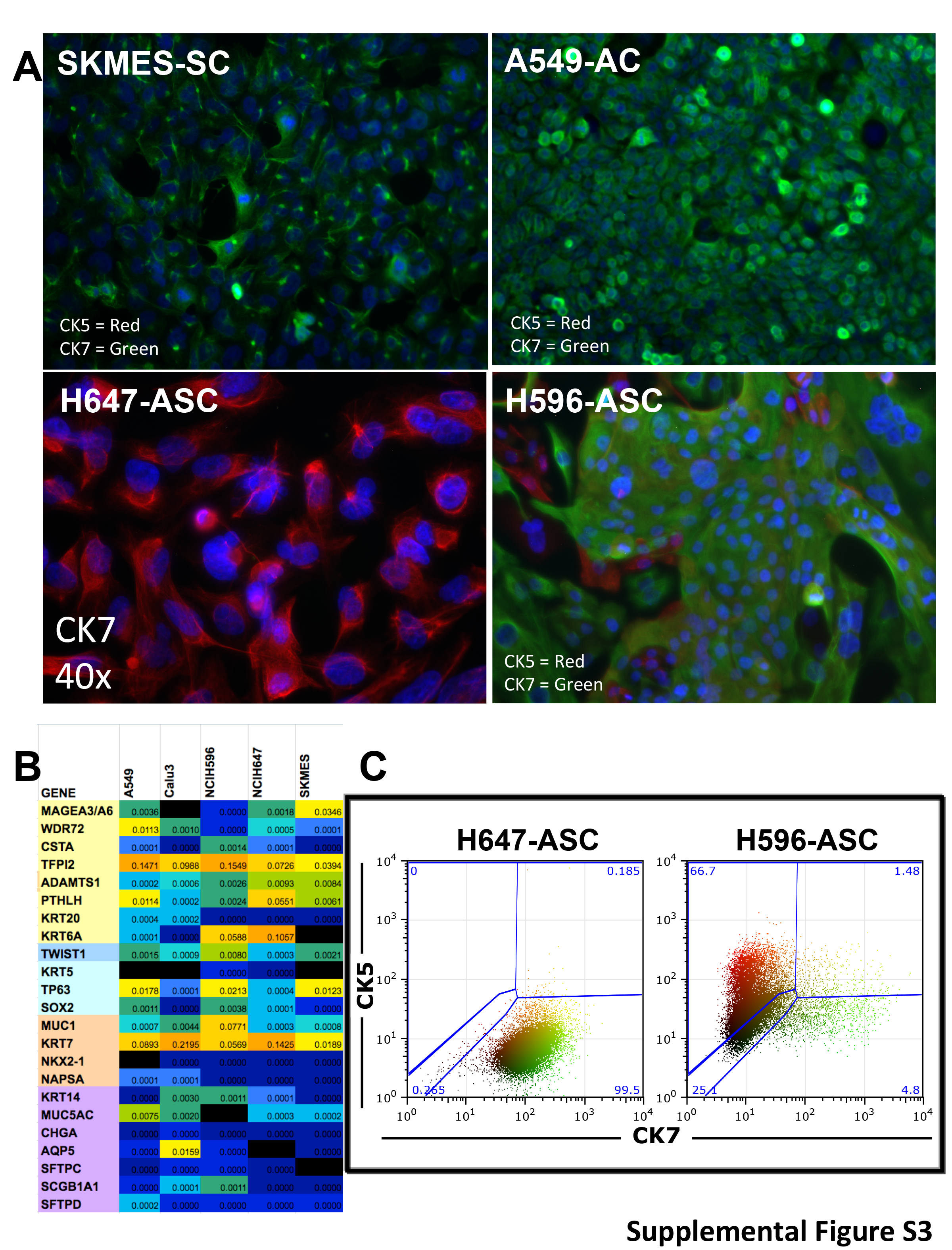

Supplement: Figure S3 — Double staining of ATCC cell lines for cytokeratins 5 and 7. Fixed permeablized monolayers of SKMES (a squamous cell carcinoma line) A549 (AC), and 2 ATCC ASC lines, H596 and H647 are shown. RT-PCR Gene expression analysis of the panel of genes analyzed for the CSLC in Figure 5 is shown for 5 ATCC cell lines derived from AC, ASC, and SCC tumors (see Table S4). The flow analysis of H596 and H647 double stained for CK5 and CK7 are shown in B. (TIF) [file pone.0079456.s003.tif]

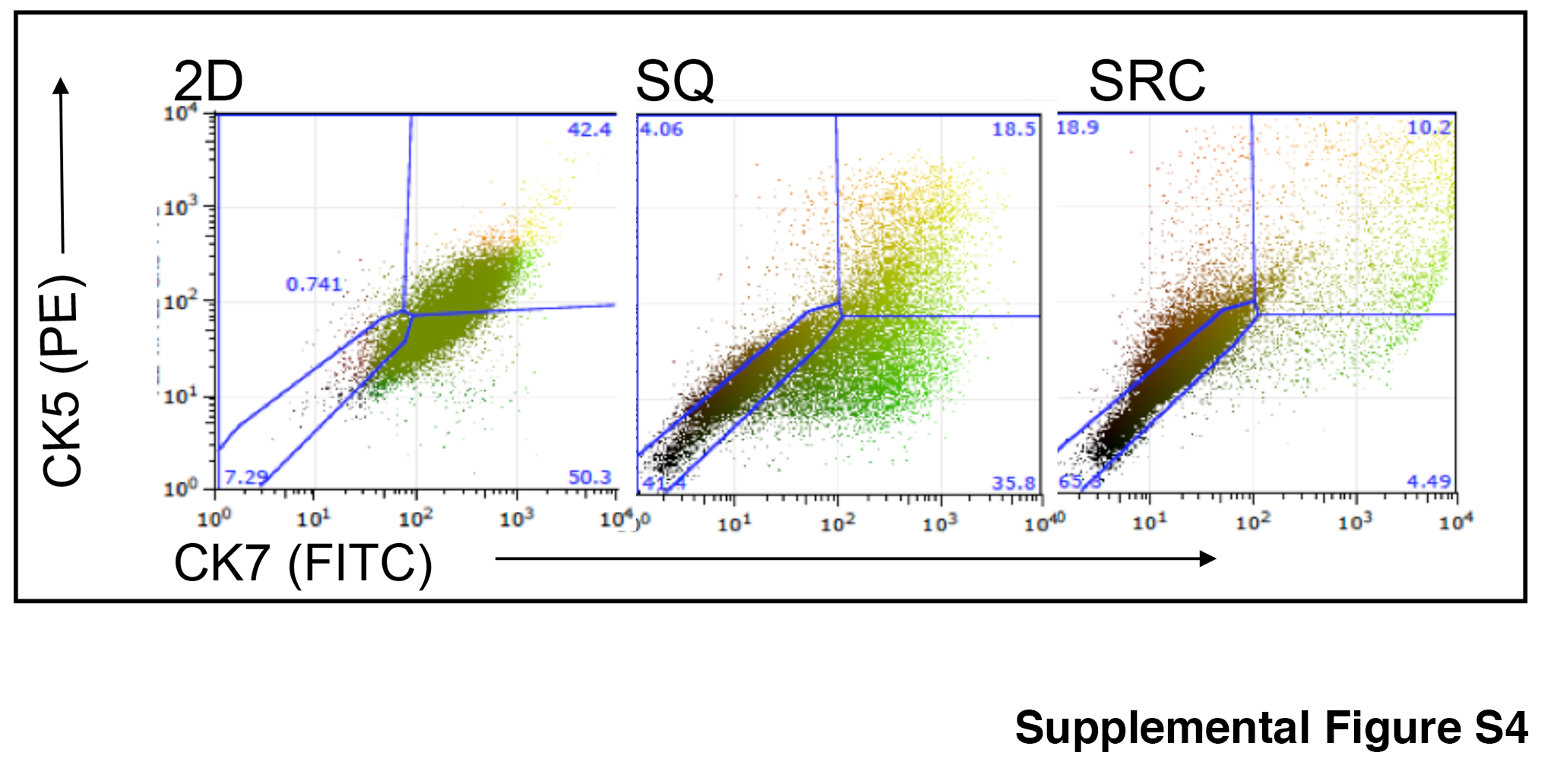

Supplement: Figure S4 — Analysis of CK5 and CK7 protein expression by flow cytometry. CK5 and CK7 protein expression was further analyzed in lung cancer derived cells by flow analysis of permeabilized LUCA22 monolayer cells double stained for CK5 and CK7 (2D). For comparison, human tumor cells isolated from subcutaneous (SQ) or sub-renal (SRC) implanted LUCA22 cells after 20 weeks in vivo are shown. Xenografts were dispersed to single cells for analysis and contain both stromal (CK7-/CK5-) and tumor cells. (TIF) [file pone.0079456.s004.tif]

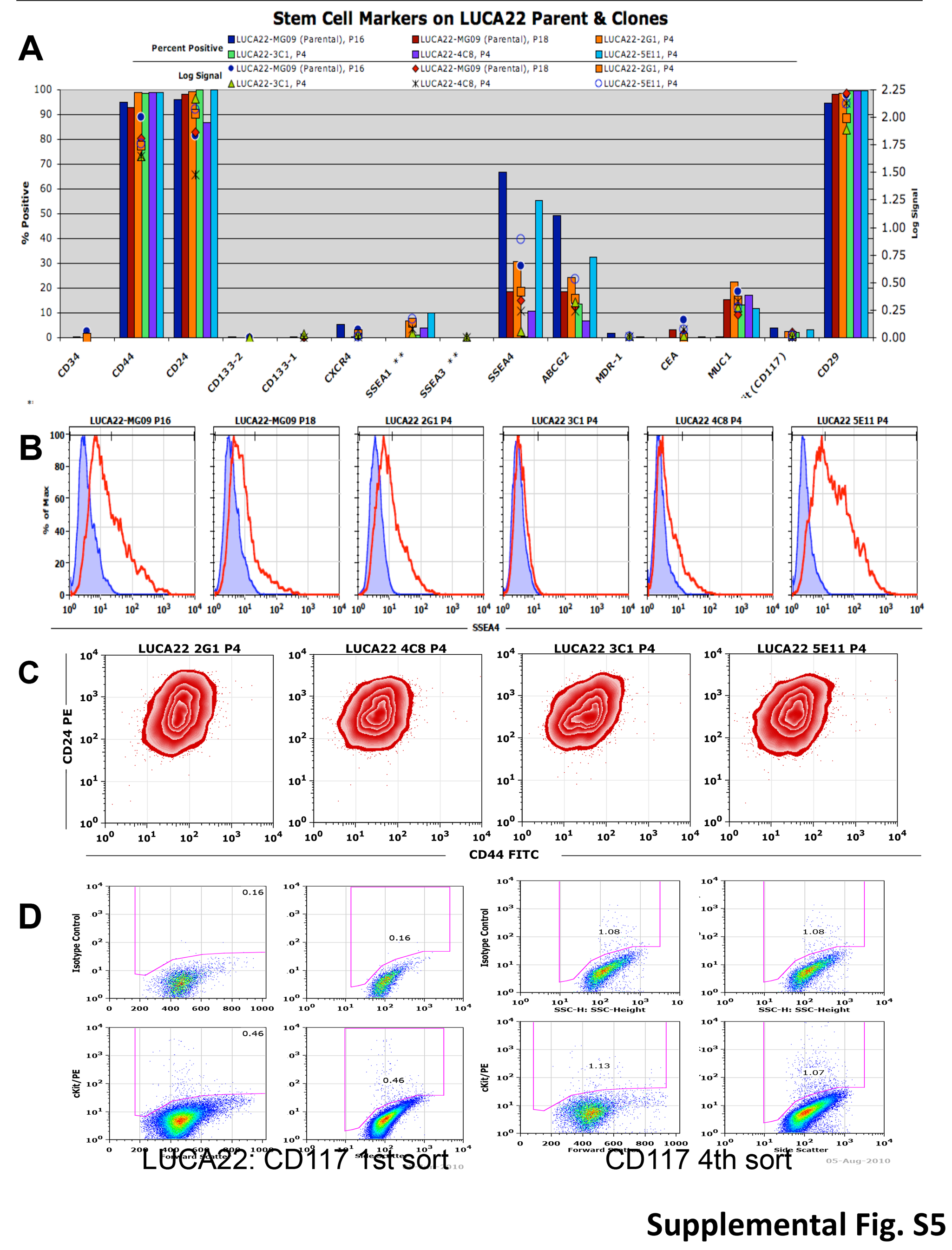

Supplement: Figure S5 — Flow analysis of cell surface proteins in 5 clones. Five randomly selected LUCA 22 clones were analyzed for expression of cell surface proteins using tagged antibodies and flow. These were compared to the parental LUCA22 line (A). The biggest variability was seen in SSEA4 expression shown as separate histograms in B. Double stain of 4 clones for CD24 and CD44 are shown in C. The diagrams in D show staining for CD117 (& side scatter) after 1 bulk sort and after the 4th successive sort of the population. The CD117 remains a minority population. (TIF) [file pone.0079456.s005.tif]

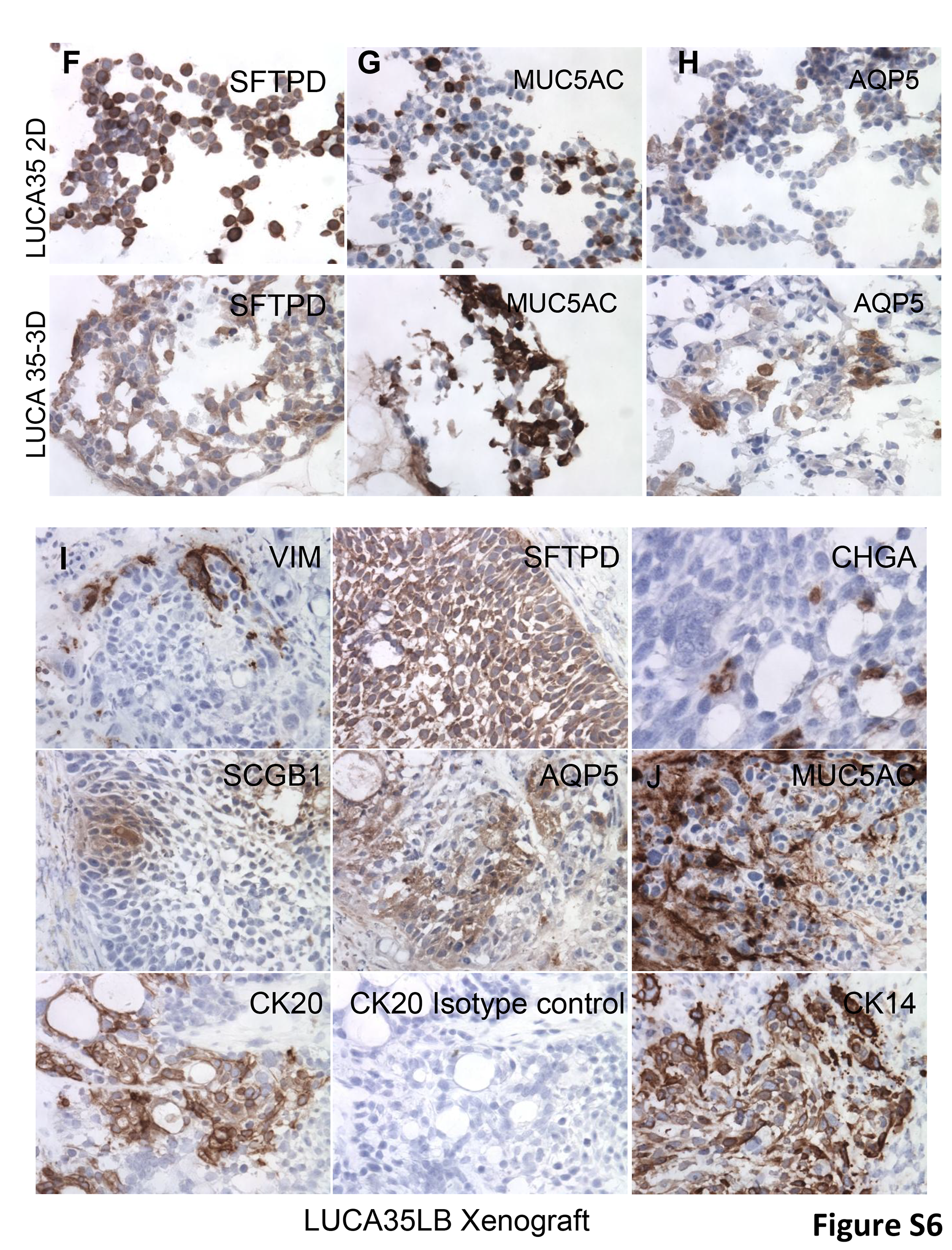

Supplement: Figure S6 — The LUCA35 cells and xenografts express multiple lung cell markers. Cells were grown in the usual medium (LUCA35 2D, F-H top) in Matrigel with differentiation medium (LUCA35 3D, F-H bottom) or in vivo as xenografts (9 panels in I). Frozen sections were stained as indicated. All isotype control stains on adjacent sections were negative (see CK20 and isotype control section, bottom center for an example). (TIF) [file pone.0079456.s006.tif]
