# Supplementary material for: Isolation of Cancer Stem Like Cells from Human Adenosquamous Carcinoma of the Lung Supports a Monoclonal Origin from a Multipotential Tissue Stem Cell
Source: PLoS One. 2013 Dec 4;8(12):e79456. doi: 10.1371/journal.pone.0079456 (PMC3850920; doi:10.1371/journal.pone.0079456)
Supplement: Methods S1 — CSLC selection and expansion from NSLC tumor tissue. LUCA CSLC characterization. Differentiation in vitro. (DOC) [file pone.0079456.s007.doc]

**Supplementary Methods S1, Mather, et al.**

- ***CSLC selection and expansion from NSLC tumor tissue***
- Lung cancer tissues were obtained through the National Disease Research Interchange. Institutional IRBs approved the protocols for tissue acquisition and use, and appropriate informed consent was obtained from patients. The lung cancer derived cell lines were established and master and working cell banks prepared and characterized.
- The defined conditions appropriate for human fetal lung tissue stem/progenitor cells [20] were optimized empirically for isolation and growth of cancer tissue as previously described [21]. Tissue was minced with scissors then disassociated using collagenase/ dispase in serum free medium in a Stomacher ^TM^ until the tumor tissue was in small clumps of cells. Cell clump were filtered through 100 uM Nytex ^TM^ to remove membranous material and undigested large clumps, and washed 3-5 times in medium. Cells were plated onto plates coated with the indicated attachment factor in F12/DMEM medium supplemented with the indicated growth factors (see Table S1). Serum free conditions were derived that enrich for, and allow expansion of, a small population of cells from ASC and AC lung tumors (Table S1). Serum free conditions were derived that enrich for, and allow expansion of, a small population of cells from ASC and AC lung tumors (Table S1). Medium was changed and cells were fed every second day and dying cells removed during the selection period. When the selected cells had expanded to fill 70-90% of the dish subculture was performed at a 1:4 split, every 5-7 days, as required to maintain cultures in a sub-confluent state. Cells were banked at passage 3-6 and used for experiments between passage10 and 35. Interestingly, these conditions do not support the isolation of cells from squamous cell carcinomas (SCC) of the lung. Addition of serum to the hormone supplements or supplementation with high levels of serum resulted in a non-tumorigenic population of cells with limited growth potential in vitro and the properties of stromal cells. Stromal cells were grown in F12/DMEM supplemented with hormones and 1% FBS, or-10% FBS, (as shown in Table S1) expanded and banked at passage 2-5. These cultures were used before passage 10. The stromal cells have a fibroblastic appearance in 10% FBS and are not tumorigenic when implanted at 5x10^5^ under the sub-renal capsule of an immunodeficient mouse. and could not be carried indefinitely in culture.
- The STR analysis used to uniquely identify the CSLC lines and their tumors of origin are shown in Table S2. Characteristics of the tumors and CSLC are described in Table S3. The properties of ATCC lines used for comparison are described in Table S4. LUCA22 cells were subjected to two rounds of *in vitro* limiting dilution cloning in 96 well plates in defined growth medium with 50% self-conditioned medium. Wells were checked visually for wells containing a single cell. Approximately 33% of 480 wells contained a single cell. Twenty-seven clones were randomly picked, expanded, and frozen. Of these, 8 clones were expanded for further study.
- ***LUCA CSLC characterization***
- The cultured cells were selected in serum-free conditions adapted from those used to establish newborn rat lung cells [20]. Hormone and growth factor additions and concentrations are described in Table S1. This medium will select and expand a small proportion of epithelial cells from both adenocarcinoma (AC) (LUCA 32, LUCA33) and adenosquamous carcinoma (ASC) (LUCA22, LUCA35) tissues, but not from squamous carcinoma tissue. Master and working cell banks were frozen at passages 3-4 and 6-8, respectively.
- ***Differentiation in vitro.***
- To study differentiation in vitro we used a 3 dimensional (3D) defined culture system modified from a differentiation culture system described by Delgado, et al. (34). For co-culture, Stromal cells and CSLC cultures were detached with trypsin-EDTA (Invitrogen) and collected by centrifuge. CSLC and stroma were mixed at different ratios from 1:1 to 1:10 to determine the optimal starting ratio for cultures. The cells were counted using a hematocytometer, then combined in an x/y ratio (CSLC: stromal cells) and pelleted by centrifugation at 1000 rpm for 5 minutes. The resulting pellet was resuspended in 50 ul F12/DMEM medium, mixed with 5 ul Matrigel, and then dispersed at 200ul/well into a 24 well plate. An optimal innoculum of: LUCA22, 10^3^ vc/well; stromal cells, 10^4^ vc/well was used for the experiments reported here. Following inoculation, the culture plate(s) was placed in a 37˚C, 5%CO_2_ humidified incubator for 1 hour, then overlaid with “differentiation medium”. Medium was changed on day 7 and thereafter twice per week. For media and hormone concentrations see Table S1. In co-cultures the CSLC rapidly proliferated and became the predominant cell in the cultures as assessed by IHC of sectioned organoids and flow analysis of separated cells.
