# Supplementary material for: Isolation of Cancer Stem Like Cells from Human Adenosquamous Carcinoma of the Lung Supports a Monoclonal Origin from a Multipotential Tissue Stem Cell
Source: PLoS One. 2013 Dec 4;8(12):e79456. doi: 10.1371/journal.pone.0079456 (PMC3850920; doi:10.1371/journal.pone.0079456)
Supplement: Results S1 — Cytokeratin staining of ATCC control cell lines. Analysis of LUCA22 clones. Use of double stains for SC and AC. (DOCX) [file pone.0079456.s008.docx]

- **Supplementary Results S1, Mather, et al.**
- **Cytokeratin staining of ATCC control cell lines.**
- As a control we stained several ATCC lung cancer cell lines (for derivation see Supplemental Table S4), including 2 ASC lines. Only the 1 of the ASC lines obtained from the ATCC, H596 stained for both CK5 and CK7, with CK5 staining predominant (Figure S3 A). The ATCC A549 and SKMES cell lines were both negative for CK5 with abundant (A549) or low levels of punctate (SKMES) CK7 staining, even though SKMES is derived from a squamous cell carcinoma. Flow cytometric analysis of CK5 / CK7 double labeled cells confirmed the expression patterns seen in staining. Genomic expression data was in good agreement with CK7 expressed in all lines and CK5 only expressed in ASC. (Figure S3 B,C)
- **Analysis of LUCA22 clones.**
- Tumorigenicity and metastasis. All eight of the LUCA22 clones selected and expanded were tumorigenic in xenograft models. Four of the eight clones were randomly selected for cell surface marker analysis. All of these clones were uniformly highly positive for CD44, CD24, and CD29. The binding to SSEA4, ABCG2, and Muc1 was lower and variable in extent, but uni-modal for all the lines (e.g. Supplemental Figure S1 B). Double label for CD24 and CD44 again demonstrated that a single population bound both antibodies (Figure S1 C). CD133 was absent in all clones. While CD117 (c-kit) binding [46] was low in all lines we were unable to increase the percentage of CD117 by selecting for high binding cells through 4 successive sorts (Supplemental Figure S5 D), again suggesting a single population of cells.
- ***Use of double stains for SC and AC***
- All primary xenografts and metastases were positive for the markers thyroid transcription factor 1 (TTF1- gene designation NKX2-1) and napsin A (NAPSA), which are found in 73% and 83%, respectively, of lung ACs and are negative in most squamous cell carcinomas and most ACs of other tissues [29]. Cells that were positive for CK5 also had positively stained nuclei for p63, consistent with a squamous phenotype of these cells (Figure S1). Interestingly a pellet of the LUCA22 cells used for implantation showed very uniform staining of cell nuclei for TTF1 with only a few scattered cells (<1%) having napsin A staining. All of the metastases showed a mosaic pattern of staining for adeno- and squamous- carcinoma. All of the xenografts derived from the eight clones grown showed regions staining with the napsin/TTF1 double stain for adenocarcinoma and regions staining for the p63/ CK5 double stain for squamous carcinoma. The stained cells for these two (napsin/TTF1 and p63/CK5 stains -Figure 2A) seem distinct but are in adjacent regions of the tumor. One clonally derived xenograft was double stained for CK5 and CK7, also staining distinct but adjacent regions of the tumor (Figure 2B).
- Both patient and xenograft tissues showed broad staining with an antibody to stem cell factor (SCF) with more restricted staining for it’s ligand c-kit (CD117) (Supplemental Figure S2A). Dual staining with anti-ALDH1A1 and CK5 showed ALDH1 staining in both the squamous and the adeno- areas of the tumor, although the ALDH1 staining was less extensive in the CK5 positive cells (Supplemental Figure S2 A). Metastases also stained positively for ALDH1 (Supplemental Figure S2 B). ALDH1A1 activity was confirmed on the LUCA22 cell line using the ALDEFLUOR^®^ assay, although only a portion of the activity was neutralized by DEAB, an ADLH1-specific inhibitor (Supplemental Figure S2C)
- Interestingly, the patient tumor and LUCA22 and LUCA35 xenograft tissues were vimentin positive in areas (Figure 1, Figure 6E, Figure S6). Xenograft tumors derived from LUCA22 also stained positive for lineage markers of epithelial cells (e.g. CK18), neuroendocrine cells, and pneumocytes. As shown in Figure 6 for LUCA22 xenografts, and Supplemental Figure S6 for LUCA35 xenografts, most cells were positive for vimentin and surfactant protein D (*SFTPD*), with subsets of cells positive for aquaporin 5 (*AQP5*) and mucin 5A (MUC5A), and a few scattered cells positive for chromagranin A (*CHGA*) and Clara cell specific protein (*SCBG1A1*). Staining of CK14 can be clearly seen to sort to basal cells, but not adjacent epithelial like cells in some areas of the xenograft (Figure 7 lower right, box).
