# Supplementary material for: Isolation of Cancer Stem Like Cells from Human Adenosquamous Carcinoma of the Lung Supports a Monoclonal Origin from a Multipotential Tissue Stem Cell
Source: PLoS One. 2013 Dec 4;8(12):e79456. doi: 10.1371/journal.pone.0079456 (PMC3850920; doi:10.1371/journal.pone.0079456)
Supplement: Table S1 — Media supplements for selective growth and differentiation of lung tumor derived cells. (DOCX) [file pone.0079456.s009.docx]

- **Supplemental Tables**
- **Table S1.** Media supplements for selective growth and differentiation of lung tumor derived cells

| - **Components** | - **CSLC growth** | - **CSLC- 3D differentiation** | - **Stromal lines** |
| --- | --- | --- | --- |
| - F12/DMEM nutrients with 1.2 gm/l Bicarbonate / 5% CO2 Trace elements | - yes | - yes | - yes |
| - Fetal bovine serum | - no | - no | - yes: 1-10% |
| - hr-Ins: 5ugm/ml hr-Tf: 5ugm/ml | - yes | - yes | - yes |
| - hr-EGF: 5ng/ml Ethanolamine 10e-6M P-Ethanolamine 10e-6M Selenium 2.5e-8M Tri-iodothyronine 10e-12M | - yes | - yes | - no |
| - hr-Heregulin 5nM Forskolin 1uM Hydrocortisone 10e-9M bFGF 5ng/ml (L35 only) Glucagon 50ng/ml | - yes | - yes | - no |
| - **B-27 Supplements*** | - no | - yes | - no |
| - **N-2 Supplements*** | - no (L35 ) | - yes | - no |
| - Fibronectin coat | - yes | - no | - no |

- * B-27 supplements – Gibco, life technologies, Ref.#17502-04. N-2 supplements - Gibco, life technologies, Ref.#17504-044
