# Supplementary material for: Isolation of Cancer Stem Like Cells from Human Adenosquamous Carcinoma of the Lung Supports a Monoclonal Origin from a Multipotential Tissue Stem Cell
Source: PLoS One. 2013 Dec 4;8(12):e79456. doi: 10.1371/journal.pone.0079456 (PMC3850920; doi:10.1371/journal.pone.0079456)
Supplement: Table S2 — STR analysis of lung cancer-derived cell lines. (DOCX) [file pone.0079456.s010.docx]

- **Table S2.** STR analysis of lung cancer-derived cell lines

| - **Sample** | - **Passage** | - **Amelo** | - **CSF 1PO** | - **D13S 317** | - **D16S 539** | - **D18S 51** | - **D19S 433** | - **D21S 11** | - **D2S 1338** | - **D3S 1358** | - **D5S 818** | - **D7S 820** | - **D8S 1179** | - **FGA** | - **THO1** | - **TPOX** | - **vWA** |
| --- | --- | --- | --- | --- | --- | --- | --- | --- | --- | --- | --- | --- | --- | --- | --- | --- | --- |
| - ***Tumor 22*** | - **NA** | - **X** | - **12, 13** | - **12, 14** | - **12, 13** | - **12, 16** | - **14** | - **30** | - **17, 23** | - **16, 17** | - **11, 13** | - **8, 10** | - **14, 15** | - **22, 23** | - **8, 9.3** | - **8, 11** | - **16** |
| - **LUCA22** | - **P 11** | - **X** | - **13** | - **14** | - **12,13,14** | - **12, 16** | - **14** | - **30** | - **17, 23** | - **17** | - **11** | - **8, 10** | - **14, 15** | - **22, 23** | - **8, 9.3** | - **8, 11** | - **16** |
| - **LUCA22** | - **P 47** | - **X** | - **13** | - **14** | - **13, 14** | - **12** | - **14** | - **30** | - **17, 23** | - **17** | - **11** | - **8, 10** | - **14, 15** | - **22, 23** | - **8, 9.3** | - **8, 11** | - **16** |
| - **LUCA22 4C8** | - **P 7** | - **X** | - **13** | - **14** | - **12, 14** | - **12, 16** | - **14** | - **30** | - **17, 23** | - **17** | - **11** | - **8, 10** | - **14, 15** | - **22, 23** | - **8, 9.3** | - **8, 1** | - **16** |
| - **LUCA22 2G1** | - **P 6** | - **X** | - **13** | - **14** | - **12, 13** | - **11, 12** | - **14** | - **30** | - **17, 23** | - **17** | - **11** | - **8, 10** | - **14, 15** | - **22, 23** | - **8, 9.3** | - **8, 11** | - **16** |
| - **LUCA22 3C1** | - **P 6** | - **X** | - **13** | - **14** | - **12, 14** | - **11*,* 12, 13,19.2** | - **14** | - **30** | - **17, 23** | - **17** | - **11** | - **8, 10** | - **14, 15** | - **22, 23** | - **8, 9.3** | - **8, 11** | - **16** |
| **LUCA22 5E11** | - **P 6** | - **X** | - **13** | - **14** | - **13, 14** | - **12** | - **14** | - **30** | - **17, 23** | - **17** | - **11** | - **8, 10** | - **15** | - **22, 23** | - **8, .3** | - **8, 11** | - **16** |
| - ***Tumor 32*** | - **NA** | - **X, Y** | - **10, 12** | - **8** | - **10, 12** | - **14, 17** | - **14** | - **28, 32.2** | - **22, 23** | - **16, 17** | - **11, 12** | - **9, 12** | - **8, 13** | - **20.2, 22, 24** | - **6, 7** | - **8** | - **18, 19** |
| - **LUCA32** | - **P 2** | - **X, Y** | - **10,12** | - **8** | - **10, 12** | - **14, 17** | - **14** | - **28,32.2** | - **22, 23** | - **17, *20*** | - **11, 12** | - **9, 12** | - **8, 13** | - **20.2, 22, 24** | - **6, 7** | - **8** | - **18, 19** |
| - **LUCA33** | - **P 5** | - **X** | - **10,11** | - **8,13** | - **9,1 3** | - **12** | - **14** | - **28,31.2** | - **17, 24** | - **15,17** | - **13, 14** | - **10** | - **13, 14** | - **22** | - **8, 9.3** | - **8,11** | - **16** |
| - ***Tumor 35*** | - **NA** | - **X** | - **12** | - **12,13** | - **12** | - **12, 13** | - **13, 14** | - **30, 31** | - **24, 25** | - **14, 15** | - **12** | - **10** | - **12, 15** | - **24, 25** | - **8** | - **8,11** | - **15, 17** |
| - **LUCA35** | - **P 13** | - **X** | - **12** | - **12** | - **12** | - **12, 13** | - **13, 14** | - **30, 31** | - **24, 25** | - **14, 5** | - **12** | - **10** | - **12, 15** | - **24, 25** | - **8** | - **8, 11** | - **15, 17** |
| - ***Tumor 11*** | - **NA** | - **X, Y** | - **10,11** | - **8,11** | - **9, 11** | - **13, 18** | - **14, 15** | - **30,32.2** | - **17, 19** | - **18** | - **11, 12** | - **8, 9** | - **13, 14** | - **23, 25** | - **7, 9.3** | - **8, 11** | - **17, 19** |
| - **LUCA11** | - **P 10** | - **X, Y** | - **10,11** | - **8,11** | - **9, 11** | - **13, 18** | - **14, 15** | - **30,32.2** | - **17, 19** | - **18** | - **11, 12** | - **8, 9** | - **13, 14** | - **23, 25** | - **7, 9.3** | - **8, 11** | - **17, 19** |
| - ***Tumor 36*** | - **NA** | - **X, Y** | - **11,12** | - **12,13** | - **10, 12** | - **15, 18** | - **13,14** | - **29,31.2** | - **18, 25** | - **14, 17** | - **10, 12** | - **9, 12** | - **12, 13** | - **22, 24** | - **9, 6.3** | - **8** | - **15, 16** |
| - **LUCA36** | - **P 6** | - **X, Y** | - **11,12** | - **12,13** | - **10, 12** | - **15, 18** | - **13, 14** | - **29,31.2** | - **18, 25** | - **14, 17** | - **10, 12** | - **9, 12** | - **12, 13** | - **22, 24** | - **6, 9.3** | - **8** | - **15,1 8** |
