# Supplementary material for: Isolation of Cancer Stem Like Cells from Human Adenosquamous Carcinoma of the Lung Supports a Monoclonal Origin from a Multipotential Tissue Stem Cell
Source: PLoS One. 2013 Dec 4;8(12):e79456. doi: 10.1371/journal.pone.0079456 (PMC3850920; doi:10.1371/journal.pone.0079456)
Supplement: Table S3 — Characteristics of Lung Tumor-derived cell lines. (DOCX) [file pone.0079456.s011.docx]

- **Table S3.** Characteristics of Lung Tumor-derived cell lines

| - **CSLC Line** | - **Tumor Type** | - **Stage** | - **Mutations*** |
| --- | --- | --- | --- |
| - **LUCA22** | - Lung (left lower lobe) adenosquamous carcinoma | - poorly differentiated pT2 pN0 M0 | - *KRAS* G12V |
| - **LUCA35** | - Lung (rt lower lobe) adenosquamous carcinoma | - moderately differentiated **+**: CK5, CK7, p63 **-** : TTF-1, CD56 | - NA |
| - **LUCA37-STROMA** | - Lung (rt lower lobe) squamous cell carcinoma | - G3 Poorly differentiated pT2a pN0 | - NA |
| - **LUCA32** | - Lung (left lower lobe) adenocarcinoma | - G3 Poorly differentiated | - *KRAS* G12V |
| - **LUCA33** | - Lung (rt upper lobe) adenocarcinoma | - G3 Poorly differentiated | - *KRAS* G12C |
| - **LUCA11-STROMA** | - Squamous cell carcinoma | - Poorly differentiated T2 N0 MX | - NA |
| - **LUCA36-STROMA** | - Lung (left upper lobe) adenocarcinoma | - G3 Poorly differentiated pT3 pN2 | - NA |

- NA- analysis not available
