# Supplementary material for: Isolation of Cancer Stem Like Cells from Human Adenosquamous Carcinoma of the Lung Supports a Monoclonal Origin from a Multipotential Tissue Stem Cell
Source: PLoS One. 2013 Dec 4;8(12):e79456. doi: 10.1371/journal.pone.0079456 (PMC3850920; doi:10.1371/journal.pone.0079456)
Supplement: Table S4 — Characteristics of ATCC lung cancer cell lines. (DOCX) [file pone.0079456.s012.docx]

**Table S4.** Characteristics of ATCC lung cancer cell lines

| - **ATCC cell line** | - **Cell Type** | - **ATCC # / (Year established)** | - **Mutations*** |
| --- | --- | --- | --- |
| - **A549** | - Lung carcinoma | - CCL-185 (1972) | - None found* |
| - **SKMES** | - Squamous cell carcinoma from pleural effusion | - HTB-58 (1975) | - None found* |
| - **Calu-3** | - Adeno carcinoma from pleural effusion | - HTB-55 (1975) | - None found* |
| - **H647** | - Adenosquamous carcinoma from pleural effusion Stage 3A | - CRL-5834 (1983) | - None found* |
| - **H596** | - Adenosquamous carcinoma from Tumor mass | - HTB-178 (1983) | - None found* |

- Mutation analysis from: [*http://www.sanger.ac.uk/cosmic*](http://www.sanger.ac.uk/cosmic)
