# Supplementary material for: Isolation of Cancer Stem Like Cells from Human Adenosquamous Carcinoma of the Lung Supports a Monoclonal Origin from a Multipotential Tissue Stem Cell
Source: PLoS One. 2013 Dec 4;8(12):e79456. doi: 10.1371/journal.pone.0079456 (PMC3850920; doi:10.1371/journal.pone.0079456)
Supplement: Table S5 — “RT2 qPCR Primer Assay” probe catalog numbers. (DOCX) [file pone.0079456.s013.docx]

- **Table S5.** “RT^2^ qPCR Primer Assay” probe catalog numbers

| - **Gene** | - **Protein Name** | - **Catalog Number** |
| --- | --- | --- |
| - MAGEA3/A6 | - melanoma-associated antigen 3/A6 | - PPH72421A-200 |
| - WDR72 | - WD repeat-containing protein 72 | - PPH14406A-200 |
| - CSTA | - cystatinA | - PPH19620A-200 |
| - TFPI2 | - tissue factor pathway inhibitor 2 | - PPH02580A-200 |
| - ADAMTS1 | - A disintegrin & metalloproteinase , thrombospondin 1 | - PPH01149A-200 |
| - PTHLH | - parathyroid hormone-related protein | - PPH02141A-200 |
| - KRT20 | - cytokeratin 20 | - PPH10312A-200 |
| - KRT6A | - cytokeratin 6A | - PPH17955E-200 |
| - TWIST1 | - Twist-related protein 1 | - PPH02132A-200 |
| - KRT5 | - cytokeratin 5 | - PPH02625E-200 |
| - TP63 | - tumor protein 63 | - PPH01032E-200 |
| - SOX2 | - sex determining region Y-box2 | - PPH02471A-200 |
| - MUC1 | - mucin1 | - PPH01085A-200 |
| - KRT7 | - cytokeratin 7 | - PPH08502E-200 |
| - NKX2-1 | - NK2 homeobox 1 | - PPH00246A-200 |
| - NAPSA | - napsinA | - PPH58016A-200 |
| - KRT14 | - cytokeratin 14 | - PPH02389A-200 |
| - MUC5AC | - mucin 5AC | - PPH60210G-200 |
| - CHGA | - chromagranin A | - PPH01181A-200 |
| - AQP5 | - aquaporin 5 | - PPH16382A-200 |
| - SFTPC | - pulmonary surfactant associated protein C | - PPH07047A-200 |
| - SCGB1A1 | - Clara Cell-Specific 10Kd protein (CC10) | - PPH02860E-200 |
| - SFTPD | - pulmonary surfactant associated protein D | - PPH15147B-200 |

- Probes were all purchased from SA Biosciences
