# Supplementary material for: Isolation of Cancer Stem Like Cells from Human Adenosquamous Carcinoma of the Lung Supports a Monoclonal Origin from a Multipotential Tissue Stem Cell
Source: PLoS One. 2013 Dec 4;8(12):e79456. doi: 10.1371/journal.pone.0079456 (PMC3850920; doi:10.1371/journal.pone.0079456)
Supplement: Table S6 — Tumorigenicity of LUCA22 and clones in SRC model. (DOCX) [file pone.0079456.s014.docx]

- **Table S6.** Tumorigenicity of LUCA22 and clones in SRC model

| - Time *in vivo* wks | - LUCA 22 parental line | | | | | | - 8 Clones | | - parental line | | | | | |
| --- | --- | --- | --- | --- | --- | --- | --- | --- | --- | --- | --- | --- | --- | --- |
|  | - 5x 10e5 cells | | | | | | | | - 5x10e4 | | - 5x10e3 | | - 5x10e2 | |
|  | - Size | - Met | - Size | - Met | - Size | - Met | - Size | - Met | - Size | - Met | - Size | - Met | - Size | - Met |
| - 8 | - ++^1^ | - no |  |  |  |  | - - /++ | - no^2^ | - + |  |  |  |  |  |
| - 10 |  |  | - +/- | - no |  |  | - **+/++** | - **yes^2^** |  |  | - +/- |  | - +/- |  |
| - 11 |  |  |  |  | - +++ | - yes |  |  |  |  |  |  |  |  |
| - 12 |  |  |  |  | - ++ | - no |  |  |  |  |  |  |  |  |
| - 15 |  |  | - +++ | - yes |  |  |  |  |  |  |  |  |  |  |
| - 16 | - +++ | - yes | - +++ | - yes |  |  |  |  |  |  | - +/- | - no | - +/- | - no |
| - 17 | - +++ | - yes |  |  |  |  |  |  |  |  |  |  |  |  |
| - 18 |  |  |  |  |  |  |  |  |  |  |  |  |  |  |
| - 21 |  |  | - +++ | - yes |  |  |  |  |  |  | - + | - no | - + | - no |
| - 23 |  |  | - +++ | - yes |  |  |  |  |  |  |  |  |  |  |
| - 25 |  |  |  |  |  |  |  |  | - +++ | - yes |  |  |  |  |
| - 27 |  |  |  |  |  |  |  |  | - +++ | - yes | - ++ | - no | - + | - no |
| - 28 |  |  |  |  | - +++^3^ | - yes | - +++^3^ | - yes^3^ |  |  |  |  |  |  |
| - 29 |  |  |  |  |  |  |  |  | - +++ | - yes |  |  |  |  |
| - 31 |  |  |  |  |  |  |  |  | - +++ | - yes | - ++ | - no | - ++ | - no |

- Notes: 1. Tumor size indicated as: –, no visible tumor growth; +/-, small visible tumor … to +++, very large tumor covering most or all of top of kidney
- 2. 3 clones + parent implanted 1 clone had tumor at 8 weeks. This was continued to 28 weeks with larger tumors and metastases. The parental control cells also formed tumors at this time. The other 2 clones were re-implanted for longer times (see below) where they grew tumors.
- 3. Implanted 3 clones (n=3) and parent line: 12/12 animals grew tumors; 1/3 animals had metastases in 2 /3 clones at 8 weeks. Implanted 4 additional clones (n=3) and parent: 14/15 animals grew tumors.
